# Cryptic Diversity in Metropolis: Confirmation of a New Leopard Frog Species (Anura: Ranidae) from New York City and Surrounding Atlantic Coast Regions

**DOI:** 10.1371/journal.pone.0108213

**Published:** 2014-10-29

**Authors:** Jeremy A. Feinberg, Catherine E. Newman, Gregory J. Watkins-Colwell, Matthew D. Schlesinger, Brian Zarate, Brian R. Curry, H. Bradley Shaffer, Joanna Burger

**Affiliations:** 1 Department of Ecology, Evolution, and Natural Resources, Rutgers University, New Brunswick, New Jersey, United States of America; 2 Department of Biological Sciences, Louisiana State University, Baton Rouge, Louisiana, United States of America; 3 Museum of Natural Science, Louisiana State University, Baton Rouge, Louisiana, United States of America; 4 Division of Vertebrate Zoology, Yale Peabody Museum of Natural History, New Haven, Connecticut, United States of America; 5 New York Natural Heritage Program, State University of New York College of Environmental Science and Forestry, Albany, New York, United States of America; 6 New Jersey Division of Fish and Wildlife, Endangered and Nongame Species Program, Clinton, New Jersey, United States of America; 7 Department of Ecology and Evolutionary Biology, University of California Los Angeles, Los Angeles, California, United States of America; 8 La Kretz Center for California Conservation Science, Institute of the Environment and Sustainability, University of California Los Angeles, Los Angeles, California, United States of America; 9 Cell Biology and Neuroscience, and Environmental and Occupational Health Sciences Institute, Rutgers University, Piscataway, New Jersey, United States of America; Field Museum of Natural History, United States of America

## Abstract

We describe a new cryptic species of leopard frog from the New York City metropolitan area and surrounding coastal regions. This species is morphologically similar to two largely parapatric eastern congeners, *Rana sphenocephala* and *R. pipiens*. We primarily use bioacoustic and molecular data to characterize the new species, but also examine other lines of evidence. This discovery is unexpected in one of the largest and most densely populated urban parts of the world. It also demonstrates that new vertebrate species can still be found periodically even in well-studied locales rarely associated with undocumented biodiversity. The new species typically occurs in expansive open-canopied wetlands interspersed with upland patches, but centuries of loss and impact to these habitats give some cause for conservation concern. Other concerns include regional extirpations, fragmented extant populations, and a restricted overall geographic distribution. We assign a type locality within New York City and report a narrow and largely coastal lowland distribution from central Connecticut to northern New Jersey (based on genetic data) and south to North Carolina (based on call data).

## Introduction

In order to develop clear understandings of species and their ecologies, distributions, and conservation needs, they must first be properly identified and accurately delimited [1]. Such efforts can be complicated, however, by the presence of cryptic species – species that, due to morphological similarity, have been incorrectly included with one or more other species under a single species classification [2]. Identifying cryptic species can be difficult though, which presents taxonomic and conservation challenges. These challenges can be further exacerbated in heavily altered environments and areas where extirpations and habitat loss have led to insufficient numbers of individuals or populations for sampling. Nonetheless, a cryptic species discovery can have important implications for multiple species, including the new species itself and its cryptic congeners [1]. Further, cryptic species can be found in unexpected locales [3], and in some regions, reflect surprisingly high levels of diversity [4]. Left undetected, however, cryptic species can remain concealed among other species, which can be problematic if seemingly common or widespread nominal species actually contain hidden component species that are range-restricted, rare, or even extinct [1], [2].

Considerable effort has been given to identifying and cataloging new species, cryptic and otherwise, over the past few decades. In the case of amphibians, these efforts carry added urgency in the face of severe global declines and extinctions and also reveal strongholds of undocumented species, often in areas of tropical species richness or poorly known composition [4], [5]. In contrast, far less attention or discovery has been associated with urban areas and other highly developed or well-documented regions, especially those outside the tropics. Among anurans, for example, only two truly novel species (that is, taxa that were not previously recognized as subspecies) have been reported from the continental United States (US) and Canada since 1986 [3], [6], [7]. In this paper we describe the most recent of these, a cryptic leopard frog lineage that was first identified from the New York City region in 2012 [3]. Few examples of undescribed vertebrate diversity exist in the recent literature from highly urbanized regions and areas with well-established taxonomic infrastructures.

The species we describe here was first identified by Newman *et al.* [3] via molecular data. It constitutes the newest member of the *Rana pipiens* complex and occupies parts of the lower Northeast and mid-Atlantic US within the densely populated and heavily industrialized Interstate-95 (I-95) corridor. This is one of the largest human population centers on earth [8] and a region where endemic vertebrate species are rare. The long-term concealment and recent discovery of a novel anuran here is both surprising and biogeographically significant, and illustrates how new species can occur almost anywhere. It also raises potentially important conservation concerns: amphibians can be sensitive to disease, contaminants, and environmental perturbations, and their low vagility can be particularly problematic in fragmented and urban landscapes [9]. Also worrisome are enigmatic declines that have led to disappearances of leopard frogs from parts of the Northeast and mid-Atlantic US [10]–[13]; this includes some relatively non-urbanized coastal, suburban, and agricultural regions in southeastern New York (NY) [3], [14], southern Connecticut (CT) [11], and presumably parts of northeastern Pennsylvania (PA) where they were reported historically, but not in recent decades [15]–[20].

Here, we expand upon the initial genetic results presented by Newman *et al.* [3] to name, diagnose, and describe the new species. We present several lines of supporting evidence, but focus on bioacoustic signals and molecular data. We also provide a brief history of relevant taxonomic confusion within the *R. pipiens* complex, comparisons to similar species, and information on distribution, ecology, and conservation status.

### Taxonomic Overview

Although one of the most well-known and best-studied amphibian groups on earth, the *R. pipiens* complex has long been a source of taxonomic uncertainty and nomenclatural debate in eastern North America [21]–[27]. Our work resolves some of this confusion. In this section we review relevant background information to provide appropriate context for our discovery.

The unsettled taxonomic history of the *R. pipiens* complex spans several centuries and has been fueled largely by a lack of scientific consensus and changing species concepts across those years. This has led to numerous synonyms and conflicting species frameworks over time [28]. Ultimately, however, only two species, *R. sphenocephala* and *R. pipiens*, received lasting consideration and taxonomic recognition in the east [26], [29]. *Rana sphenocephala*, the southern leopard frog, has a reported range from extreme southeastern NY to Florida (FL) and west from Texas to Iowa [30]. *Rana pipiens*, the northern leopard frog, ranges from eastern Canada, New England, and the northern mid-Atlantic, west to the Pacific Coast states and British Columbia [30]. These two species are generally parapatric along the US East Coast [29], [30], although Pace [26] reported one possible example of sympatry from Bronx County, NY (but see Klemens *et al.* [31]).

Much of the historical discord and confusion surrounding the *R. pipiens* complex can be traced to the Northeast and mid-Atlantic US [26], [27], [32], especially the greater New York City metropolitan area [11], [33], [34] (referred to hereafter as the NY/NJ-metro area and defined to include southwestern CT, southeastern NY, New Jersey [NJ], and extreme eastern PA). This relatively small region has been associated with longstanding ambiguity regarding leopard frogs, including the type locality of *R. pipiens* itself [7], [34], [35] and as many as five different species names over the past 250 years [7], [33].

In 1936, Kauffeld [35] attempted to reconcile some of this confusion. He did so by noting the possibility of a third, centrally occurring and unnamed “form” of leopard frog in the NY/NJ-metro area, between the recognized East Coast ranges of *R. sphenocephala* and *R. pipiens* at that time. Kauffeld [33] later combined his own examinations with subspecies descriptions by Cope [36] and putative type localities for *R. pipiens* to conclude that three distinct species did occur across the Northeast and mid-Atlantic US. He classified the northernmost species as *R. brachycephala* and reassigned *R. pipiens* – the binomial typically associated with the northernmost species – to his proposed central species (occupying much of the NY/NJ-metro area and mid-Atlantic region with extensions south along the coastal plain and west to Texas); *R. sphenocephala* was maintained as the southernmost species. Despite acknowledging the potential taxonomic confusion and backlash this could cause, Kauffeld [33] proposed these changes to reflect his conclusion that the type locality for *R. pipiens* fell within southeastern New York, where his reported central species occurred, not the northernmost species.

Kauffeld's three-species framework and taxonomic changes received some initial recognition [37]–[39] but did indeed face considerable scrutiny over time and failed to garner lasting support [23]–[25]. His proposals also provided the impetus for several studies that led to more conservative taxonomic frameworks, including the predominant mid-20^th^ Century single-species interpretation that classified all North American leopard frogs as *R. pipiens* [24], [40], [41]. This determination was based on inconsistent differences among purported species and successful cross-breeding experiments with frogs from distant geographies [28], [42]. Several decades later, relying primarily on morphology and bioacoustics, Pace [26] presented a detailed treatment of the *R. pipiens* complex that returned to a two-species arrangement in the eastern US, echoing arrangements prior to Kauffeld's work [43]–[45]. This included *R. sphenocephala* (referred to as *R. utricularia* by Pace) to the south, and *R. pipiens* to the north, with a species boundary centered in the NY/NJ-metro area. Pace's arrangement remained largely intact over subsequent decades, particularly across the eastern US.

Occasional discussion of distinct populations, potential intergradation, and cryptic species in the NY/NJ-metro area continued after Kauffeld [33], but remained largely speculative [11], [46], [47]. More recently, however, advances in molecular methods utilizing nuclear and mitochondrial markers have allowed for increasingly sophisticated species delimitations and analyses of phylogenetic and population genetic relationships. Initial molecular work by Newman *et al.* [3] demonstrated this, suggesting that an undescribed cryptic leopard frog lineage, termed *R*. sp. nov., does indeed occur between populations of *R. sphenocephala* and *R. pipiens* in the NY/NJ-metro area. They also reported mitochondrial data showing this species to be most closely related to the pickerel frog, *R. palustris*, a morphologically distinct and readily identifiable species [29], rather than to *R. sphenocephala*, the species to which it had been included based on morphological similarity; nuclear data regarding interspecific relationships were inconclusive.

In retrospect, the long history of taxonomic and nomenclatural confusion in the NY/NJ-metro area was likely due to the unrecognized presence of a cryptic species occurring in close proximity to several similar congeners. For example, in the Philadelphia region – an area replete with historical confusion and variation reported among leopard frogs [26], [27], [48] – all four regional spotted congeners are now known to occur; *R. pipiens*, *R. palustris*, *R*. sp. nov., and *R. sphenocephala* each occur in succession along a narrow 90-km west-to-east transect between Berks County, PA and Burlington County, NJ [20], [49].

## Materials and Methods

### Ethics Statement

The species described here was discovered during research activities conducted under an Institutional Animal Care and Use Committee Protocol (IACUC) from Rutgers University (#07-024). Additional field work and collection of the holotype specimen occurred under New York State Collect or Possess permit #969 (to MDS) in compliance with Yale University IACUC protocol #2012-10681.

### Taxonomic Note

We briefly point to an area of unresolved taxonomic debate within the herpetological community. This debate centers on use of the historical genus name *Rana* versus a recently proposed replacement name, *Lithobates*, which has been applied to a number of North American ranid frog species [50]. Given that this issue still remains largely unsettled, we have followed the conservative taxonomic practice of continuing to use *Rana* for all North American ranid frogs, including the *R. pipiens* complex.

### Morphology

**Figure 1:**
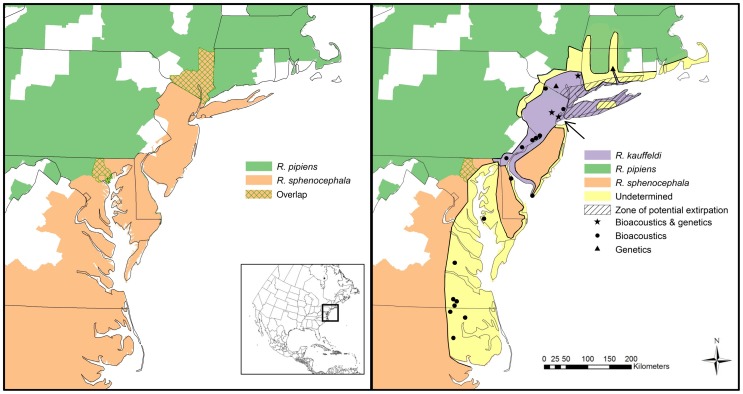
Leopard frog distributions in the Northeast and mid-Atlantic US. Left: currently recognized IUCN (2012) range maps for *R. pipiens* (green) and *R. sphenocephala* (red) with areas of potential overlap (hatched). Right: newly interpreted distributions for all three leopard frog species including *R. kauffeldi*. Symbols indicate known *R. kauffeldi* populations and purple shading depicts areas where our field work has confirmed the occurrence of *R. kauffeldi*. Yellow shading indicates areas of less intensive examination and sampling; *R. kauffeldi* may occur in these areas based on habitat and proximity to known populations. Potential sympatry is also possible in the yellow shaded areas, with *R. sphenocephala* (from Long Island southward), or *R. pipiens* (north and west of Long Island). The type locality for *R. kauffeldi* is indicated by an arrow.

Fieldwork to collect an adult male holotype was conducted in Richmond County, NY. The specimen was preserved in 10% neutral-buffered formalin, transferred to 70% ethanol and deposited at the Yale Peabody Museum of Natural History (YPM). We collected morphometric measurement data from 283 specimens, including the holotype (YPM 13217) and 282 other museum specimens across four species (*R*. sp. nov., *R. sphenocephala, R. pipiens*, and *R. palustris*), 30 US counties, seven eastern states, and Quebec, Canada (Table S1). When genetic data were not available to confirm species identification, we used a combination of morphology and location to classify preserved specimens based on our knowledge of species habitat preferences and distributions (Fig. 1). Straight-line measurements were taken to the nearest 0.01 mm with Mitutoyo Digimatic calipers. We measured 13 characters, 11 of which follow Napoli [51]: snout-vent length (SVL; anterior end of snout to posterior end of urostyle), head length (HL; anterior end of snout to occiput), head width (HW; at widest part of the head), eye diameter (ED; at widest point of eye), tympanum diameter (TD; at widest point of tympanum), foot length (FOL; tip of fourth toe to heel), eye to naris distance (END; anterior eye to naris), naris to snout distance (NSD; naris to anterior end of snout), thigh length (THL; anterior knee to posterior urostyle), internarial distance (IND; closest distance between nares), and interorbital distance (IOD, closest distance between the eyes). We also include shank length (SL; knee to heel) following Heyer *et al.* [52] and dorsal snout angle (DSA; [arcsine ((HW/2)/HL) ×2) following Lemmon *et al.* [6].

We looked for univariate differences in species morphology using boxplots and one-way ANOVAs followed by Tukey HSD post-hoc pairwise comparisons. We used discriminant function analysis (DFA) to examine variation in multivariate space and determine which variables best discriminated among species. This was followed by a MANOVA to look for multivariate differences among species, and then Tukey HSD post-hoc pairwise comparisons. Because body size varied substantially among specimens, we removed this effect in our statistical analyses by using the residuals of a regression of snout-vent length on each morphometric variable. Foot length was not available for some specimens (*n* = 19), reducing the number of frogs with complete measurements to 264. Thus, we omitted these specimens from our DFA. All analyses were conducted in R, v. 2.15.2 and v. 3.0.2 [53], including package MASS.

We also examined color and patterning differences between leopard frog species. We compared dorsal spots (number of spots and percent dorsal area coverage) between the new species and its closest morphological congener, *R. sphenocephala*, following Platz [54]. For spot coverage, we imported images of both species (*R.* sp. nov., *n* = 22; *R. sphenocephala*, *n* = 18) into ArcMap 10.0 [55] and digitized polygons representing the dorsum and each spot as viewed from directly above in order to calculate the proportion of the dorsal surface covered by spots. We examined both variables using boxplots and t-tests (α = 0.05) to look for species differences. We also conducted several categorical comparisons between *R*. sp. nov. and *R. sphenocephala*, including 1) dorsal spot shape (round or elongate), 2) snout spot (present or absent), and 3) skin color (three color categories). We categorized a dorsal spot as ‘elongate’ if it was at least 2.5 times longer than wide at its widest point, but excluded eyelid spots from this analysis because the curvature of the eye made them difficult to assess. Lastly, we compared pigmentation on the posterior dorsal surface of the femur (thigh) among specimens of *R.* sp. nov., *R. sphenocephala*, and *R. pipiens*. This character was previously used to distinguish leopard frogs in regions where *R.* sp. nov. occurs [24], [32]. We follow Moore [24] in referring to it as the “reticulum” and recognize two alternate states: light (light ground color with dark spots) or dark (dark ground color with light spots). All specimens used in spot and color comparisons are listed in Table S1. All photo vouchers were deposited at YPM.

### Genetic Analysis

Following the methods described in Newman *et al.* [3], we extracted genomic DNA from a liver sample obtained from the holotype. We sequenced the ND2 and 12S–16S regions of the mitochondrial genome, including intervening and flanking tRNAs (1444 bp), and the nuclear genes neurotrophin-3 (NTF3, 599 bp), tyrosinase (Tyr, 557–585 bp), Rag-1 (647–683 bp), seven-in-absentia (SIA, 362–393 bp), and chemokine receptor 4 (CXCR4, 550 bp). PCR products were sequenced at Beckman Coulter Genomics (Danvers, MA, USA). All sequences generated in this study were uploaded to GenBank (accession number of hologenetypes: JX867559-JX867563). Data from the present study were added to the Newman *et al.* [3] data set, and Bayesian phylogenetic analyses were run in MrBayes 3.1 [56], [57] for each locus following the analyses described in Newman *et al.* [3] to verify the species identity of the holotype.

### Bioacoustic Analysis

We recorded calls of the new species with an Olympus DS-40 digital voice recorder and Sennheiser MKE 400 directional microphone at a sampling rate of 44.1 kHz and 16-bit sampling size. We converted files to.wav format using Roxio Sound Editor (Sonic Solutions, Novato, CA, USA) and analyzed calls with RAVEN Pro v. 1.4 [58] using the following settings: spectrogram FFT length 2048, Hanning window size 1024, amount of overlap between FFT samples 90, and power spectrum FFT length 2048. We analyzed calls from three populations (two in Richmond County, NY; one in Bergen County, NJ). For comparison, we also recorded and analyzed calls from four congeners using these same methods unless otherwise stated (Table S2); these included *R. sphenocephala*, *R. pipiens*, *R. palustris*, and an acoustically similar species outside the leopard frog complex, *R. sylvatica*. We examined two populations of *R. sphenocephala* (Middlesex Co., NJ and Burlington Co., NJ), one population of *R. pipiens* (Columbia Co., NY), one population of *R. palustris* (Suffolk Co., NY), and three populations of *R. sylvatica* (Queens Co., NY, Suffolk Co., NY, and Larimer Co., Colorado). We did not collect frogs used in our call analysis, but deposited call vouchers at YPM (Table S2).

We measured seven variables: call length (CL; time from beginning to end of a single call), call rate (CR; based on time between starts of successive calls), call rise time (CRT; time from call start to maximum amplitude), call duty cycle (CDC; call length/[call length + time to next call start]), pulse number (PN; number of pulses in a call), pulse rate (PR; based on time between start of first and last pulse), and dominant frequency (DF; frequency of highest energy in a call). We mostly follow parameters and terminology from Cocroft and Ryan [59] but follow Lemmon *et al.* [6] for CDC and PN. We derived trait averages from four consecutive calls per individual unless otherwise noted (Table S2). For the purposes of this study, we examined only the primary mating call of each species, defined as the advertisement call by Heyer *et al.* [52]. This approach provided a clear means for comparing species and minimized confusion presented by secondary call signals. Thus, all secondary repertoires were considered to fall outside the scope of our objectives and were not analyzed here. We compared call differences between species using the same univariate and multivariate statistical procedures described for our morphological analyses. Call rate and call length are frequently correlated with water temperature, so we adjusted these two parameters to a common water temperature of 14°C for our statistical analyses following Lemmon *et al.* [6]. We used regression equations from *R.* sp. nov. in place of *R. pipiens* and *R. palustris* because both species were recorded at only one site each under a single temperature regime, and thus lacked sufficient variation for us to generate their own species-specific regression equations.

### Nomenclatural Acts

The electronic edition of this article conforms to the requirements of the amended International Code of Zoological Nomenclature, and hence the new names contained herein are available under that Code from the electronic edition of this article. This published work and the nomenclatural acts it contains have been registered in ZooBank, the online registration system for the ICZN. The ZooBank LSIDs (Life Science Identifiers) can be resolved and the associated information viewed through any standard web browser by appending the LSID to the prefix “http://zoobank.org/”. The LSID for this publication is: urn:lsid:zoobank.org:pub:2E7F07A6-19B1-4352-B5B7-A227A93A37CD. The electronic edition of this work was published in a journal with an ISSN, and has been archived and is available from the following digital repositories: PubMed Central and LOCKSS.

## Results

### Diagnosis and Description

#### *Rana kauffeldi* sp. nov

urn:lsid:zoobank.org:act:149ED690-FA7D-4216-A6A1-AA48CC39B292.

#### Holotype

**Figure 2:**
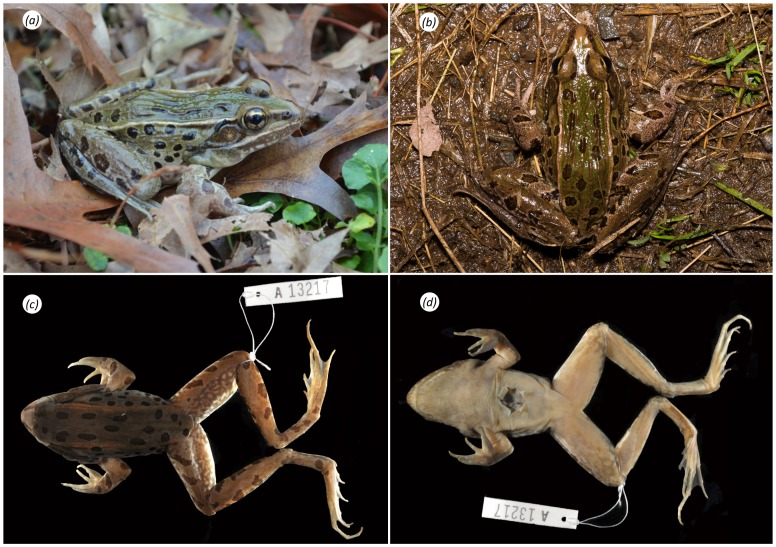
Photographs of *Rana kauffeldi* sp. nov. holotype (YPM 13217). Male frog presented live: (a) whole body, dorsolateral view and (b) dorsal view; and preserved: (c) dorsal view and (d) ventral view. Photographs taken by BRC (a), BZ (b), and GWC (c–d).

YPM 13217, adult male (Fig. 2, Table 1), collected from Bloomfield region, Richmond County (Staten Island), NY, United States, on 15 November 2011, by B. R. Curry.

**Table 1:** Mean morphological parameters for four species of *Rana*.

		*R. kauffeldi*	*R. sphenocephala*	*R. pipiens*	*R. palustris*
Variable	Holotype	(*n* = 160)	(*n* = 46)	(*n* = 47)	(*n* = 30)
SVL	50.03	57.16±9.81	57.92±10.03	58.24±9.76	51.73±7.80
range		20.34–85.07	42.47–84.1	42.25–83.23	31.53–66.24
HL	18.87	18.59±2.81	19.92±2.98	18.26±2.79	17.42±2.25
range		11.53–27.49	14.77–28.02	13.30–25.75	11.06–21.05
HW	15.73	18.87±3.40	18.09±3.25	18.66±3.11	17.41±2.46
range		9.95–26.60	12.65–25.83	13.75–25.75	10.69–22.23
ED	6.29	4.69±1.01	5.65±1.49	6.29±1.04	4.21±1.21
range		1.19–7.80	2.82–9.51	3.74–8.52	2.72–7.43
TD	4.18	4.81±0.91	4.68±0.84	4.43±0.85	3.92±0.55
range		1.77–7.15	3.15–6.54	3.00–6.92	2.65–5.00
FOL	43.52	48.35±8.12	49.73±7.96	50.65±7.51	44.28±5.75
range		17.79–65.35	36.57–69.84	38.67–66.82	28.97–56.62
END	3.81	3.98±0.66	4.74±0.97	4.38±0.64	4.00±0.63
range		2.25–5.97	3.40–7.44	3.27–6.08	2.62–5.30
NSD	3.19	3.78±0.78	4.02±0.84	4.59±0.91	3.52±0.52
range		1.20–6.31	2.69–7.04	3.11–7.11	2.55–4.74
THL	29.09	27.24±4.90	30.26±6.49	30.42±5.98	27.07±4.18
range		15.61–41.81	20.12–48.22	20.87–45.27	17.75–35.69
IND	3.53	3.95±0.80	3.75±0.74	4.29±0.80	3.79±0.77
range		1.18–6.05	2.15–5.60	2.87–6.11	2.71–5.38
IOD	3.55	4.19±0.84	3.68±0.72	3.40±0.68	3.63±0.76
range		1.88–6.72	2.57–5.24	2.26–4.67	2.41–5.32
SL	28.65	31.98±5.32	33.60±6.36	34.89±5.68	30.91±4.66
range		18.65–46.96	20.91–49.27	25.89–48.46	19.76–40.79
DSA	0.86	1.06±0.10	0.94±0.08	1.07±0.08	1.05±0.07
range		0.76–1.32	0.79–1.12	0.93–1.22	0.94–1.20

All measurements in mm, unless otherwise noted. Mean includes ± standard deviation (SD). Thirteen characters are listed as follows: snout-vent length (SVL), head length (HL), head width (HW), eye diameter (ED), tympanum diameter (TD), foot length (FOL), eye-to-naris distance (END), naris-to-snout distance (NSD), thigh length (THL), internarial distance (IND), interorbital distance (IOD), shank length (SL), and dorsal snout angle (DSA, radians). Nineteen frogs were omitted from FOL measurements (see Table S1). Note: the above values come from unadjusted (raw) data whereas size-corrected residual values were used in all other morphometric analyses.

#### Paratypes

YPM 13559, subadult male (paragenetypes: GenBank accession numbers JN227403, JN227458, JN227127, JN227180, JN227236, JN227348, JN227292) and YPM 13560, adult male (paragenetypes: GenBank accession numbers JN227404, JN227459, JN227128, JN227181, JN227237, JN227349, JN227293); both collected from Wangunk Meadows in Portland, CT by T. Mahard and M. Blumstein on 15 September 2010; genetically confirmed within the same clade as the holotype [3].

#### Referred material

YPM 13920, juvenile (GenBank accession numbers JN227377, JN227432, JN227102, JN227155, JN227209, JN227321, JN227265); collected as an egg by J. A. Feinberg from the type locality on 27 March 2009 (hatched in captivity and raised *in situ* within a field enclosure on Long Island, NY, for a separate research project); genetically confirmed within the same clade as the holotype [3]. AMNH 121857–121858, juveniles; collected from type locality on 3 August 1984 by P. R. Warny and E. Johnson.

#### Etymology

The specific epithet is a patronym in recognition of Carl F. Kauffeld who studied the *R. pipiens* complex in the NY/NJ-metro area and concluded that three distinct species, including an undocumented central species, occurred there.

#### Common Name

We propose the common name ‘Atlantic Coast Leopard Frog’ for this species.

#### Synonymy

Given the complex nomenclatural history of leopard frogs in the NY/NJ-metro area, we searched for potential synonyms within the range of *R. kauffeldi* before assigning a binomial and identified five candidates: *R. pipiens* Schreber [60], *R. halecina* Daudin [61], *R. utricularius* Harlan [48], *R. virescens virescens* Cope [36], and *R. brachycephala* Cope [36] as elevated to species rank by Kauffeld [33]. Based on our review and commentary by Lavilla *et al.* [62] and Frost [7], we determined that none of these candidates has clear unequivocal support or the precise locality information or type specimens necessary to warrant assignment to the new species. Most recently, Frost *et al.* [50] proposed *Lithobates pipiens* as a systematic replacement for *Rana pipiens*, but the type locality was not changed, and, as noted earlier, disagreements in the herpetological community as to the utility and appropriateness of *Lithobates* remain largely unsettled at this time.

We include *R. pipiens* as a synonym because its type locality has been restricted to various parts of the NY/NJ-metro area where *R. kauffeldi* occurs [7], [34], [35], [63], [64]. However, given the lack of precision, geographic consensus, or a physical type specimen, Pace [26] designated a neotype from Tompkins County in central NY (UMMZ 71365). We follow Pace, and thus consider *R. pipiens* to be removed from further geographic consideration, and also agree with Smith [65] and Pace [26] that the frog illustrated by Schreber [60] most resembles the northernmost species, not the species described here. Thus recircumscription of the geographic range of *R. pipiens* is unwarranted and, despite the confusion and numerous synonymies from the NY/NJ-metro area, no other synonym conclusively warrants resurrection. We also refer briefly to Lavilla *et al.* [62] and point out that *R. halecina* was introduced to translate a Swedish name but was not intended as a scientific name. Further, it comes only from an observation and lacks an explicit type locality or type specimen.

#### Diagnosis

**Figure 3:**
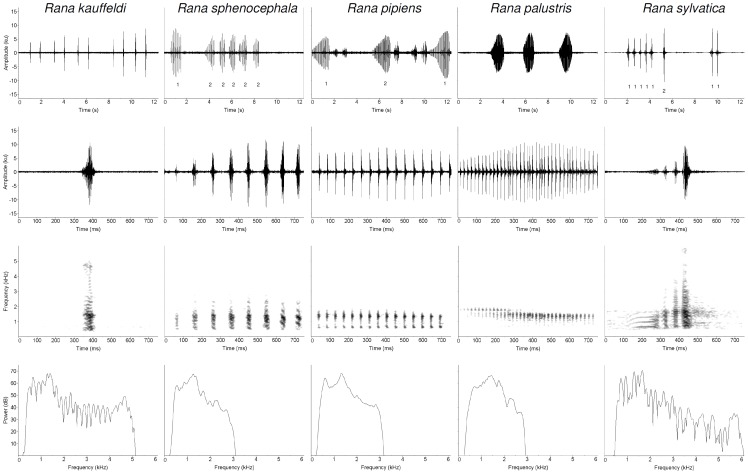
Primary (advertisement) calls of five *Rana* species from the study region. Species include *R. kauffeldi* (column 1), *R. sphenocephala* (column 2), *R. pipiens* (column 3), *R. palustris* (column 4), and *R. sylvatica* (column 5). Depicted individuals were recorded within 8°C of each other at 10.0, 11.0, 18.0, 15.0, and 10.1°C, respectively. Row 1 shows waveforms of primary call sequences (12 s scale) (note: *R. pipiens* contains secondary grunts). Rows 2 and 3 show single-call waveforms and spectrograms, respectively (750 ms scale). Row 4 shows power spectra for each single call. Numbers assigned to waveforms in row 1 indicate and identify different individuals. Format adapted from Lemmon et al. [6].

*Rana kauffeldi* is morphologically similar to *R. sphenocephala* and *R. pipiens*, but distinguishable by 1) advertisement call (Fig. 3, Table 2; Figs. S2 and S4), 2) genetics [3], 3) habitat (see *Distribution*), 4) geographic distribution (Fig. 1), and 5) a combination of morphological characters (Table 1; Figs. S1 and S3).

**Table 2:** Mean primary (advertisement) call parameters for five species of *Rana*.

	*R. kauffeldi*	*R. sphenocephala*	*R. pipiens*	*R. palustris*	*R. sylvatica*
Variable	(*n* = 13)	(*n* = 8)	(*n* = 4)	(*n* = 11)	(*n* = 9)
CL (ms)	55.81±10.86	534.45±158.69	1905.42±352.45	1429.90±237.24	205.89±86.27
range	33.25–71.25	364.50–796.00	1604.50–2409.33	1130.00–1825.00	85.25–330.25
CR (calls/s)	1.34±0.46	1.38±0.39	0.07±0.01	0.19±0.09	1.72±0.77
range	0.70–2.35	0.96–1.90	0.06–0.08	0.09–0.33	0.68–2.85
CRT (ms)	31.52±7.66	422.64±159.81	1299.65±223.73	856.40±218.27	169.85±80.75
range	18.00–47.25	212.33–636.00	1001.5–1519.67	595.33–1267.67	57.50–289.75
CDC	0.07±0.02	0.71±0.05	0.14±0.03	0.28±0.10	0.39±0.24
range	0.05–0.10	0.62–0.79	0.10–0.16	0.12–0.41	0.06–0.66
PN	1.00	7.85±1.05	38.83±7.76	61.15±9.10	2.51±0.67
range	1.00	6.25–9.50	29.50–48.33	47.50–78.67	1.50–3.33
PR (pulses/s)	0	13.57±3.53	19.79±1.92	42.52±5.41	7.79±1.17
range	0	9.77–17.82	17.75–22.38	30.26–47.96	6.19–9.23
DF (Hz)	1383.11±116.41	1214.86±226.09	1174.91±103.91	1264.43±251.86	1426.79±214.89
range	1211.23–1593.48	785.98–1476.58	1098.20–1327.90	947.50–1937.97	947.47–1679.60

Seven bioacoustic characters are listed as follows: call length (CL), call rate (CR), call rise time (CRT), call duty cycle (CDC), pulse number (PN), pulse rate (PR), and dominant frequency (DF). Mean includes ± standard deviation (SD). Note: the above values come from unadjusted (raw) data; in all other bioacoustic analyses CL and CR were corrected to a common temperature of 14°C, following Lemmon *et al*. [6].

The advertisement call is a single-noted unpulsed ‘chuck’ (Video S1) that is distinct from the pulsed ‘ak-ak-ak’ of *R. sphenocephala* and the snore-like calls of *R. pipiens* and *R. palustris*. The quivering ‘quack’ of *R. sylvatica* is superficially similar but consists of discrete bouts of 2–4 rapidly pulsed notes that are never accompanied by secondary ‘groans’ as occasionally emitted by *R. kauffeldi*. Although sympatric with *R. kauffeldi*, *R. sylvatica* is morphologically and genetically distinct and typically calls from smaller canopied wetlands and forested pools whereas *R. kauffeldi* usually calls from larger, open-canopied wetlands.

**Figure 4:**
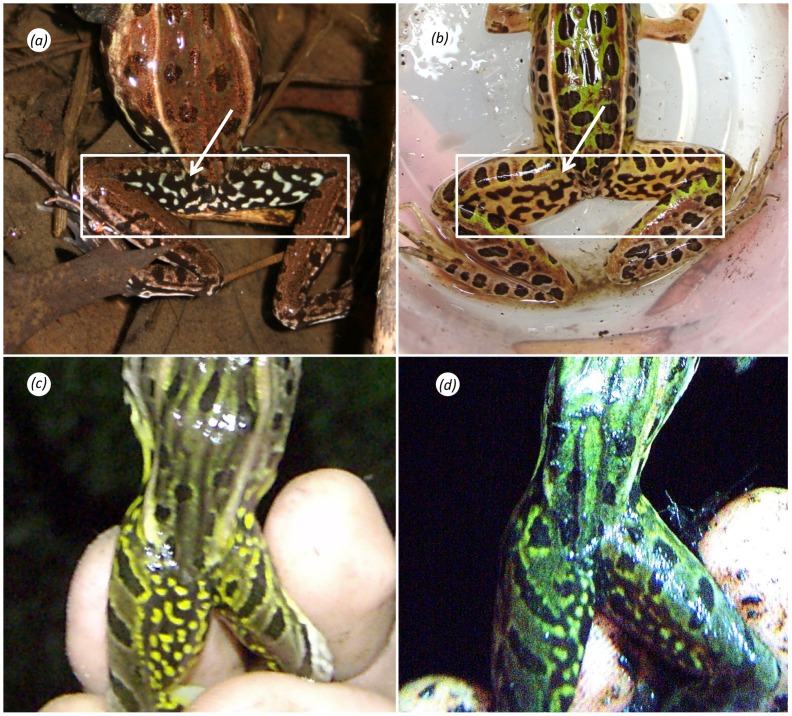
Reticulum shading patterns. Examples include (a) dark state, *Rana kauffeldi* (YPM 14143); (b) light state, *R. sphenocephala* (YPM 14097); (c) *R. kauffeldi* yellow variant (YPM 13767); (d) *R. kauffeldi* green variant (YPM 14025). Photographs taken by E. Kiviat (a), M. Cram (b), and BRC (c, d).

Adult male *R. kauffeldi* possess very large, laterally paired external vocal sacs that distinguish them from all similar congeners except *R. sphenocephala*. Additionally, *R. kauffeldi* has a dark femoral reticulum (Fig. 4*a*) whereas northeastern populations of *R. sphenocephala* and *R. pipiens* typically have a light reticulum (Fig. 4*b*). This diagnostic was 100% consistent in *R. kauffeldi* from NY and NJ (*n* = 27) and *R. pipiens* from the northeastern US and Canada (*n* = 46), and was 88.6% consistent in *R. sphenocephala* from NJ (*n* = 35). The diagnostic value of this character may be limited to northern regions, however, as Moore [24] noted that leopard frogs predominantly exhibit a dark reticulum across portions of the Southeast where *R. sphenocephala* is broadly distributed.

*Rana kauffeldi* may be further distinguished from *R. sphenocephala* by a tympanic spot that is typically duller, less well-defined, and rarely pure white (as in *R. sphenocephala*); from *R. pipiens* by a light spot in the center of the tympanum that is often small and faint (but occasionally absent); and from *R. palustris* by pale inner thighs without deep yellow coloration and round, unaligned dorsal spots.

#### Description

Body moderate and robust; head longer than wide. Dorsal outline of snout acuminate; lateral snout profile round. Nares dorsolaterally oriented, slightly protuberant, around two-thirds closer to tip of snout than anterior corner of eye. Canthus rostralis distinct and angular; loreal region steep and slightly concave. Eyes large and protuberant; diameter slightly less than combined eye-to-naris and naris-to-snout distances. Internarial distance nearly equal to eye-to-naris distance. Tympanum distinct and relatively large (>65% diameter of the eye); bordered dorsally and posteriorly by faint supratympanic fold. Distinct dorsolateral fold runs uninterrupted from posterior eye to pelvic insertion of femur. Forearms relatively short and robust; unwebbed fingers; relative length III>I>II>IV. Fingers lack fringes; tips rounded without expansion; subarticular tubercles small, round, and moderately prominent. No palmer tubercles appear present. First finger slightly swollen at base with faint nuptial pad; all other fingers slender. Hindlimbs relatively long, moderately robust; thigh and shank length nearly equal. Relative toe lengths IV>V>III>II>I; toes have rounded tips without expansion; subarticular tubercles small, round, and prominent. Inner tarsal fold connects tarsus to large, distinct, elliptical, elevated inner metatarsal tubercle. Indistinct, small outer metatarsal tubercle faintly evident. Toe IV very long and slender; toe V slightly fringed; webbing present between all toes; webbing formula **I**1 – 2**II**1^+^ – 2⅓**III**1^+^ – 3^+^ **IV**3 – 1**V** following Savage [66]. Skin on dorsum smooth with several raised folds running between and parallel to dorsolateral folds. Flanks, thighs, and shanks smooth. Ventral surface mostly smooth with papillae-like granulation on groin and thighs. Large, distinct, paired lateral external vocal sacs.

#### Color in life

In photographs taken before preservation, dorsal ground color of holotype varies from mint-gray in bright lighting (Fig. 2*a*) to light olive green in darker conditions (Fig. 2*b*). Medium to dark brown spots irregularly distributed across dorsum and lateral body; more elongate or barred on the limbs. Distinct black postorbital patch encompasses dorsal and posterior tympanum along the supratympanic ridge. Labial margins slate gray with light mottling and distinct ivory stripe above the upper margin; terminates under the tympanum (continues to anterior forearm in females). Dark canthal band runs from snout tip through the nare and iris, along outer edge of dorsolateral fold; terminates above the arm. On snout, inner edge of canthal band is paralleled by light brown band that continues through the eyelid to merge with a dorsolateral fold that varies from gold (Fig. 2*a*) to bronze (Fig. 2*b*) in different lighting. Iris gold with dark intrusions at corners. Vocal sac slightly darker than surrounding skin. Lower flank of holotype pale with light yellowish-green hues and smaller, lighter spots and mottles; these intrude onto ventral margins, throat, or body in some individuals. Tympanum finely granulated brown color with black flecks; central spot creamy and subtly defined in holotype; bright and well defined or entirely absent in some individuals. Reticulum and anterior ventral margin of thigh dark with distinct light flecks or mottles; off-white in holotype, occasionally bone-white (Fig. 4*a*), light yellow (Fig. 4*c*) or green (Fig. 4*d*) in some individuals. Ventral limbs of holotype pinkish-gray with scattered mottles; body pale white. Inner tarsal fold and outer metatarsal tubercle are bright white against a dark brown tarsal background; webbing pale gray.

#### Color in preservative

Generally similar to that in life with several notable distinctions. Ground color dark olive green in holotype (Fig. 2*c*) but can range from tan to dark brown in other specimens (as in paratypes YPM 13559 and 13560). Colored flecks and mottles in life appear white in preservative. Ventral body and limbs of holotype cream, light mottling behind knees (Fig. 2*d*). Dorsolateral fold of holotype rust brown (Fig. 2*c*); off-white to brown in other individuals. Tympanic spot, when present as in the holotype, typically subtle and grayish white.

### Genetics

Holotype (YPM 13217) falls within the *R. kauffeldi* clade (*R.* sp. nov. in Newman *et al.* [3]) in the mitochondrial phylogeny (results not shown). Mitochondrial and nuclear haplotypes are identical to other *R. kauffeldi* samples. As reported by Newman *et al.* [3], *R. kauffeldi* is genetically distinct from all other regionally occurring spotted ranid frogs (*R. sphenocephala, R. pipiens, and R. palustris*). The mitochondrial phylogeny suggests that *R. kauffeldi* is most closely related to *R. palustris*. Average pairwise mitochondrial sequence divergence (uncorrected p) is similar to genetic divergences between other closely related species in the *R. pipiens* complex (Newman *et al.* [3]).

### Distribution

*Rana kauffeldi* is known from three states (CT, NY, NJ) based on genetic samples [3] and seven states (NY, NJ, PA, Delaware [DE], Maryland [MD], Virginia [VA], and North Carolina [NC]) based on bioacoustic sampling reported here. The estimated range from these samples is approximately 780 km, north-to-south, from central CT to northeastern NC (Fig. 1). The range is narrow, however, east-to-west, occurs almost entirely within the densely populated I-95 corridor, and is smaller than most if not all other ranid frogs along the eastern North American seaboard. Within the presented range, we depict a core sampling area (Fig. 1, purple shading) where gaps in genetic and bioacoustic information were filled by other lines of evidence (e.g., specimens, photographs, geology, or historical literature). *Rana kauffeldi* appears to occur parapatrically in this core area. Beyond the core area, we depict an extended area of potential occurrence (Fig. 1, yellow shading) based on habitat features and proximity to known bioacoustic confirmations in DE, MD, VA, and NC. Within the yellow shading we also note the potential for sympatry with *R. sphenocephala* (in the south) and *R. pipiens* (in the north) based on genetic, bioacoustic, and specimen sampling (see *Discussion*).

*Rana kauffeldi* has a mesic distribution that is wider in the north and narrows from Trenton, NJ, to the Delmarva Peninsula. This part of the range essentially follows the Delaware River floodplain and the Atlantic Fall Line – the geologic interface between the relatively xeric Atlantic coastal plain where *R. sphenocephala* occurs, and more interior and upland regions to the west – where *R. pipiens* occurs. This species is usually abundant where it occurs, but populations in the NY/NJ-metro area tend to be disjunct and isolated from one another and often occur in highly fragmented landscapes with limited connectivity or dispersal opportunities. *Rana kauffeldi* was generally included within the range of *R. sphenocephala* prior to its discovery, but northern mainland populations from northeastern PA to central CT may have been included within *R. pipiens* instead (Fig. 1, yellow shading).

We also consider *R. kauffeldi* to have previously occurred within parts of an apparent extirpation zone that includes most of coastal NY and southern CT (Fig. 1). We used multiple lines of evidence to inform this conclusion, including historical locality information [11], [33], photographs [67]–[69], call descriptions [68], [70], personal communications (A. Sabin and F. C. Schlauch), and museum specimens (Table S1). Our assessment of museum specimen and photographs included frogs from Long Island (*n* = 27) and Bronx County, NY (*n* = 7). Based on our examination, 29 of these 34 frogs were *R. kauffeldi*. Two other individuals, from xeric parts of Long Island, NY (Suffolk County), appeared to be *R. sphenocephala* (AMNH 125956, 176153). The remaining three frogs were *R. pipiens*, two of which (AMNH 106549, 106550) came from the Bronx County site previously noted by Pace [26] and Klemens *et al.* [31], where specimens of *R. kauffeldi* (AMNH 52342, 106551–10654) were also collected historically. The third was a lone individual from western Long Island, in Queens County, NY (AMNH 36651). We also examined specimens (*n* = 9) from two presumably extirpated sites in southeastern CT (New Haven County) (Table S1). All were *R. pipiens*, but neither site is coastal or located within a bottomland riparian floodplain where *R. kauffeldi* would be expected to occur.

### Morphological Evidence

Univariate analysis recovered significant differences among 11 of 12 size-corrected characters between *R. kauffeldi* and *R. sphenocephala*, *R. pipiens*, and *R. palustris* (Fig. S1). *Rana kauffeldi* had 1) the shortest eye-to-naris distance (*F*_3,279_ = 28.41, *p*<0.0001), 2) shortest thigh length (*F*_3,279_ = 22.63, *p*<0.0001), and 3) shortest shank length (*F*_3,279_ = 27.95, *p*<0.0001) of the four species examined. *Rana kauffeldi* had 4) narrower eyes (*F*_3,279_ = 41.61, *p*<0.0001), 5) a wider head (*F*_3,279_ = 14.59, *p*<0.0001), 6) and longer interorbital distance (*F*_3,279_ = 35.02, *p*<0.0001) than *R. sphenocephala* and *R. pipiens*. *Rana kauffeldi* also had 7) a shorter head than *R. sphenocephala* and a longer head than *R. pipiens*, (*F*_3,279_ = 16.00, *p*<0.0001), 8) a longer internarial distance than *R. sphenocephala* and a shorter internarial distance than *R. pipiens* (F_3,279_ = 8.48, *p*<0.0001), 9) a larger tympanum diameter than *R. pipiens* and *R. palustris* (*F*_3,279_ = 14.42, *p*<0.0001), 10) a shorter naris-to-snout distance (*F*_3,279_ = 19.92, *p*<0.0001) than *R. pipiens*, and 11) a wider snout angle than *R. sphenocephala* (*F*_3,279_ = 32.04, *p*<0.0001). The unadjusted summary data for all 13 morphometric characters are also presented (Table 1).

In multivariate space using DFA, we found considerable morphological overlap among all four species examined (Fig. S2), but some significant differences were detected (*F*_3,260_ = 120.0, *p*<0.0001). The DFA correctly classified 78.0% of specimens (Table S3). Post-hoc Tukey's HSD tests showed all pairwise comparisons to be significantly different from one another (*p*<0.0001) except for *R. sphenocephala* and *R. palustris* (*p* = 0.9966). The first discriminant function accounted for 58.4% of the variation in the data with tympanum diameter loading most heavily, while the second function accounted for 31.4% of the variation with eye-to-naris distance having the greatest load (Table S4).

Previous studies report fewer and smaller dorsal spots among leopard frogs from areas where *R. kauffeldi* occurs [24], [32], and we found that *R. kauffeldi* indeed has fewer dorsal spots than *R. sphenocephala* (mean  = 13.18±3.22 SD vs. 20.44±4.10 SD, respectively) (*t* = −4.32, two-tailed *p*<0.001) and less dorsal surface covered by spots (mean  = 13.56%±3.29 vs. mean  = 22.13%±7.76, respectively) (*t* = −6.12, two-tailed *p*<0.0001) (Fig. S3). Dorsal spot shape also differed; only 35.71% (*n* = 42) of *R. kauffeldi* had one or more elongated spot compared to 61.16% (*n* = 67) of *R. sphenocephala* examined. Further, snout spots were present in 32.86% (*n* = 70) of *R. kauffeldi* versus 16.88% (*n* = 77) of *R. sphenocephala*. Lastly, we found considerable categorical color differences between *R. kauffeldi* (*n* = 75) (74.7% =  dark olive to mint-gray, 24.0% =  green to light brown, and 1.3% =  bright green) and *R. sphenocephala* (*n* = 94) (46.8% =  dark olive to mint-gray, 39.4% =  green to light brown, and 13.8% =  bright green). Multi-colored frogs were categorized by their lightest color.

### Bioacoustic Evidence

The unpulsed advertisement call of *R. kauffeldi* is typically emitted in evenly spaced, repeated series that can include up to 27 ‘chucks’ over 22 s. Calls were recorded at multiple locations within the type locality. Five males (YPM 14137–14140; Table S2) were recorded at the specific location where the holotype itself was heard calling and collected (but not recorded). These frogs were recorded between 2028 and 2042 h on 15 March 2012 (11°C air, 10°C water) and had the following mean characteristics: call length 60.55 ms (54.00–71.25±6.74 SD), call rate 1.10 calls/s (0.90–1.33±0.15), call rise time 33.55 ms (29.00–39.75±4.55), call duty cycle 0.07 (0.05–0.10±0.02), pulse number 1.00 (1.00±0.00), pulse rate 0, and dominant frequency 1296.30 Hz (1211.23–1421.20±85.50). Recordings from one of these frogs (YPM 14137 and 14172) were used to represent temporal and spectral features for *R. kauffeldi* in comparison to *R. sphenocephala*, *R. pipiens*, *R. palustris*, and *R. sylvatica* in Fig. 3.

We compared summary data for all *R. kauffeldi* to the four other species (Table 2). Frogs were recorded opportunistically with water temperatures ranging from 8 to 25.6°C (Table S2), reflecting the different geographies and phenologies among species. The temperature range was less variable, however, when grouped and averaged by species; *R. kauffeldi* (12.56°C±2.87 SD), *R. sphenocephala* (18.30°C±7.80), *R. pipiens* (18.00°C±0), *R. palustris* (15.00°C±0), and *R. sylvatica* (9.68°C±0.94).

Our univariate analysis revealed significant differences among species in 6 of 7 call parameters (Fig. S4). *Rana kauffeldi* had 1) a lower pulse rate (*F*_4,40_ = 293.0, *p*<0.0001) and 2) shorter call duration than all other species (*F*_4,40_ = 171.0, *p*<0.0001), and 3) a lower pulse number (*F*_4,40_ = 280.9, *p*<0.0001) and 4) a lower call rise time than all species except *R. sylvatica* (*F*_4,40_ = 85.3, *p*<0.0001). *Rana kauffeldi* also had 5) a lower call duty cycle than all species except *R. pipiens* (*F*_4,40_ = 37.8, *p*<0.0001), and 6) a call rate that was higher than *R. pipiens* and *R. palustris* and lower than *R. sylvatica* (*F*_4,40_ = 44.8, *p*<0.0001). Dominant frequency did not differ significantly among the five species (*F*_4,40_ = 2.3, *p* = 0.0744).

In multivariate space using DFA, we found clear separation in call parameters among all species (Fig. S2). The DFA correctly classified 95.6% of calls (*F*_4,40_ = 323.7, *p*<0.0001). The only classification errors were two *R. sylvatica* classified as *R. kauffeldi* (Table S5). Post-hoc Tukey's HSD tests showed all pairwise comparisons to be significantly different from one another (*p*<0.001) except for *R. kauffeldi* and *R. sylvatica* (*p* = 0.9991). Pulse rate was excluded from the DFA because *R. kauffeldi* has only one pulse per call. The first discriminant function accounted for 61.0% of the variation in the data with call rise time loading most heavily, while the second function accounted for 24.3% of the variation with call length contributing the greatest load (Table S6).

### Ecology, Behavior, and Natural History

*Rana kauffeldi* inhabits a restricted range of mesic lowland habitats that primarily includes coastal freshwater wetlands, tidally influenced backwaters, and interior riparian valley floodplains. This species is typically associated with large wetland complexes composed of open-canopied marshes, wet meadows, and slow-flowing systems with ample open upland and early-successional habitats. Aquatic conditions are usually clear, shallow, and sometimes ephemeral, with emergent shrubs or stands such as cattail, *Typha* spp., or the invasive common reed, *Phragmites australis*.

*Rana kauffeldi* begins breeding around the same time as *R. sylvatica* and *R. sphenocephala* and slightly in advance of *R. pipiens* and *R. palustris*. In NY, we have observed migratory activity on rainy nights with above-average temperatures in early February, and have documented the onset of chorusing after several days of above-average temperatures in early-to-mid March. Choruses are most consistent nocturnally, with air temperatures ranging from 10–18°C, but sustained diurnal and nocturnal chorusing is common early in the season and through the initial 2–3 week peak breeding period (late March and early April in NY), especially on warmer days. Thereafter, chorusing tapers to a more episodic nocturnal and precipitation-based regime from mid-April through early June (in NY). We have not observed opportunistic mid-summer chorusing as we and others [26], [71] have for *R. sphenocephala*, but we have observed occasional second breeding periods with the onset of cooler autumn temperatures and precipitation (late August through November).

Individuals may exhibit a limited degree of color change around a general base color that can vary widely between frogs, from light green to dark brown. Holmes [72] noted that leopard frogs (*sensu lato*) tend towards darker nocturnal shading and brighter, more vivid diurnal colors (as a putative mode of camouflage). Some degree of seasonal color change also appears to exist in *R. kauffeldi*; we often observed frogs with darker, drabber color and fainter tympanic spots in the early spring, and more vivid and varied overall color and brighter, more defined tympanic spots later in the season.

During breeding, males congregate in concentrated groups, or possible leks [26], that typically include five or more frogs, with as few as 30 cm between individuals. Males call while floating in shallows with emergent vegetation and as little as 20 cm of water. As stated by Mathewson [73], their calls are low-pitched and do not carry far. This is especially apparent in the presence of louder, higher pitched sympatric species like spring peepers (*Pseudacris crucifer*). Thus dense aggregations may have compensatory value, especially when faced with noisy conditions [74] or acoustic competition from other anurans [9], [75], [76]. Egg masses are often clustered in groups or deposited near one another. Porter [32] and Moore [77] discussed eggs and embryonic development among specimens (referred to as *R. pipiens*) from Philadelphia and NJ, respectively, that we consider *R. kauffeldi*.

Little is known about non-breeding activity or dispersal in *R. kauffeldi*, but leopard frogs have been described as being fairly terrestrial on Staten Island [73]. In our work, we observed individuals on land later in the season, but also noted periods, typically in summer and early fall, when few if any individuals could be found. Diet is not specifically known, but is presumably similar to those reported for other regional leopard frog species.

## Discussion

### Hidden Diversity in a Well-Documented Urban Region

The description of *R. kauffeldi* brings the current number of New World leopard frogs to 19 (excluding *R. palustris*) and the total number of native ranid frog species from the US mainland and Canada to 30 [7]. Despite the vast size of this area, new frog discoveries north of Mexico are infrequent, and thus geographically significant. For example, *R. kauffeldi* and the Cajun chorus frog, *P. fouquettei,* [6] are the only newly described anurans (not former subspecies) north of Mexico in nearly three decades (since 1986) [7], and *R. kauffeldi* is the first anuran from the US Atlantic coast since the New Jersey chorus frog, *P. kalmi*, was originally recognized (as a subspecies) in 1955 [7].

The specific region where *R. kauffeldi* was first identified, the New York City metropolitan area (with a type locality less than 15 km from the Statue of Liberty) is also significant. It provides an example of new species discovery, not from a tropical biodiversity hotspot or poorly studied region, but rather the glacially impacted urban Northeast; one of the most developed, heavily settled, and well-inventoried places on earth. Novel and undescribed vertebrate species are unexpected here (particularly amphibians) and thus carry considerable interest and value. The last amphibian described from NY or New England was the Fowler's toad, *Bufo fowleri*, in 1882 [78], and *R. kauffeldi* follows the northern cricket frog, *Acris crepitans*, in 1854 [79], as the seventh amphibian described from NY [7]. Several other points warrant consideration. For one, this discovery clearly demonstrates that human knowledge of the natural world remains incomplete even in the best-known locales. Second, although new frog discoveries are generally uncommon north of Mexico, they do still occur periodically. Third, the two most recent examples (*R. kauffeldi* and *P. fouquettei* [6]) are both cryptic species. Taken together, these points suggest that occasional future discoveries from well-cataloged areas may continue, but probably in the form of additional cryptic species rather than morphologically distinct taxa (which are likely already cataloged).

Although *R. kauffeldi* is a cryptic species, it is a relatively large, conspicuous, non-fossorial species nonetheless, and acoustically distinct. That it remained ill-defined and poorly documented within one of the largest population centers on earth [8] spanning eight eastern US states and several major North American cities, is rather remarkable. As a point of comparison, we consider another cryptic species group from the eastern US, the gray treefrogs *Hyla versicolor* and *H. chrysoscelis*. Despite being arboreal, smaller, and less conspicuous than leopard frogs, these two congeners were recognized as separate and distinct species nearly 50 years earlier (in 1966) by differences in their calls [7], [80].

In part, the sustained concealment of *R. kauffeldi* may have been due to its narrow and fragmented range, short and cold-season calling regime, and low frequency (less audible) call. Repeated acoustic misidentification may have also played a concealing role; many colleagues with whom we communicated recalled unusual calls from frog populations now known to be *R. kauffeldi*. Some attributed these calls to *R. sylvatica* in unusual habitats; others presumed call variation within *R. sphenocephala*. Given these examples and the generally stereotyped and species-specific nature of frog calls [4], [9] and the nuanced-but-critical role they can play in identifying species, we encourage greater scrutiny and examination of aberrant calls elsewhere, especially when encountered and heard consistently across entire populations or regions. Such efforts may reveal additional diversity, especially in areas of systematic uncertainty or contact zones where opportunities for hybridization and speciation are most likely.

### Biogeography and Distributional Relationships with Close Congeners

New species can have important biogeographic implications, particularly when they occur within intricate species groups and complex geographic regions. In the case of *R. kauffeldi*, its discovery from the Northeast and mid-Atlantic US has direct consequences for three species across eight states (Fig. 1). Its range draws entirely from two cryptic congeners, *R. sphenocephala* and *R. pipiens*. Thus, the recognized distributions of both congeners will decrease correspondingly where *R. kauffeldi* occurs alone. These changes will refine certain ecological understandings and distributional patterns too. For example, contrary to a previously defined statewide distribution in NJ, *R. sphenocephala* is now exclusively restricted to xeric habitats such as the Pine Barrens. This constitutes a considerable departure from a previous range over a wide variety of habitats and geologies to a newly defined range that conforms to the coastal distributions of many southern herpetofaunal species.

Distributional relationships vary between *R. kauffeldi* and its close congeners. The general distributions of *R. kauffeldi* and its sister species *R. palustris* (as reported in Newman *et al*. [3]) overlap broadly [29], [30], though we did not find them together in the field and noted different general habitat preferences that may keep the two species ecologically isolated. Conversely, the distribution of *R. kauffeldi* is generally parapatric with *R. sphenocephala* and *R. pipiens*, but examples of sympatry do exist with both species. Newman *et al.* [3] provided genetic evidence of sympatry without hybridization with *R. pipiens* in CT, and we viewed museum specimens noted by both Pace [26] and Klemens *et al.* [31] that suggest additional potential sympatry in northwestern NJ (*R. kauffeldi:* AMNH 35138; *R. pipiens:* AMNH 13114, 35139). We also identified areas of sympatry between *R. kauffeldi* and *R. sphenocephala* in southeastern VA from bioacoustic evidence (North American Amphibian Monitoring Program), and suspect additional overlap in southern locales. Lastly, based on museum specimens from areas where leopard frogs are now extirpated, we note several isolated examples of possible *R. sphenocephala* from xeric eastern Long Island, NY, and *R. pipiens* from Queens and Bronx Counties, NY (Table S1). Historical species composition in these areas remains unclear, however. These sparse samples may reflect natural historical populations (and potential areas of overlap with *R. kauffeldi*) or possible human introductions; isolated geographic records can suggest captive releases [3], [31], particularly in urban areas. Thus, we excluded both urban *R. pipiens* occurrences from Fig. 1.

### Delineating Complicated Historical Ranges in Heavily Modified Landscapes

Determining the distribution of new species is essential to the process of identifying and interpreting their broader biogeographic implications. In the case of cryptic species, identifying regional compositions and reassigning museum specimens can be challenging but important, especially in heavily impacted landscapes with extirpations or species overlap. In our work, leopard frogs were simply unavailable across vast landscapes due to habitat loss and extirpations. Where individuals were available, differentiating similar-looking congeners was difficult. To overcome such challenges, several strategies can provide pathways forward, including 1) using genetic and bioacoustic methods at sites where new species and their cryptic congeners still occur to delineate species and study habitats, interactions, and hybridization; 2) using genetics and morphology to identify subtle physical differences, if any, between species; and 3) applying these insights to museum specimens and extirpated locales to help assess historical compositions and distribution where populations no longer exist. These pathways (along with genetic examination of archival specimens when possible) can link genetic and bioacoustic tools with museum specimens and morphology and can also help inform future conservation strategies and range map development.

### Management and Conservation

The addition of *R. kauffeldi* to the North American faunal record and species lists of at least eight US states will have implications at various regulatory and management levels. This will include possible threatened or endangered species considerations in certain areas, and may require further assessment of the status of *R. kauffeldi* and its cryptic congeners in some of these impacted areas. It may also provide further opportunity to investigate and verify species composition and boundaries throughout different parts of the range. This may be challenging, however, especially in states where leopard frogs (*sensu lato*) already receive legal protections and in areas where multiple species are found to co-occur. Thus, reliable, field-ready characters that distinguish similar taxa, and research on potential hybridization, are key priorities. We also leave open the possibility that *R. kauffeldi* may extend farther south.

The discovery of *R. kauffeldi* has several broad conservation implications. For one, it reaffirms that refined taxonomic information is essential for implementing proper conservation measures [2], [3]. It also reinforces the critical role that basic natural history and alternative methods, such as bioacoustic techniques, can have in distinguishing potentially rare cryptic species. Lastly, it demonstrates that undocumented species can still reside in some of the most urbanized and densely inhabited parts of the world; these areas can harbor significant biodiversity and, with proper management, simultaneously protect that diversity and provide valuable educational opportunities to urban communities. The United Nations Environment Programme and US Fish and Wildlife Service's Urban Wildlife Refuge Initiative have both focused recent efforts on protecting urban biodiversity and enhancing the value and scope of urban wildlife refuges. The discovery of *R. kauffeldi* adds another important observation to the growing consensus that we must protect sensitive species where they occur, not just in pristine environments. Findings such as this also provide invaluable opportunities to highlight and enhance access for increasingly urban societies to experience new species discoveries and taxa of high conservation concern firsthand.

The overall conservation status of *R. kauffeldi* awaits further definition of distribution and habitat use and should be considered data deficient in the IUCN classification system. On-the-ground assessments, coupled with genetic and bioacoustic data, will be critical to this and allow for more complete mapping of boundaries and overlap with related taxa. If the distribution is indeed narrow and fragmented (as reported here), it may pose some cause for concern as geographically restricted species are often at risk of extinction due to demographic stochasticity [81]. Several other conservation considerations warrant mention. First, survival prospects of *R. kauffeldi* populations in the NY/NJ-metro area vary from tenuous to stable, with the most vulnerable populations being those that are small and isolated and threatened by succeeding canopy closure and development. Second, dense breeding groups and strong metapopulation structure may be essential features of *R. kauffeldi* demography, but may also represent key vulnerabilities in the face of habitat impacts. Rorabaugh [82] expressed similar concerns in noting metapopulation susceptibility, habitat impacts, and canopy closure as potential threats for *R. pipiens*. Lastly, on a broader scale, climatic events (e.g., rising sea levels, increased storm frequencies and intensities) have the ability to alter coastlines and threaten proximate low-lying freshwater wetlands and any amphibian populations therein with potentially harmful saline inundation.

Leopard frogs (*sensu lato*) have already vanished from some parts of North America [30] including several areas specifically within the northern range of *R. kauffeld* [10], [11], [13]. Some of these disappearances were likely caused by direct habitat loss or alteration, especially in urban landscapes [10], [31]. Others, however, occurred enigmatically within less-developed coastal, suburban, and semi-rural areas (Fig. 1); this includes Long Island [3], [13], the largest island in the continental US and a former leopard frog stronghold [10] where potential causes of extirpations (e.g., disease, invasive species, and contaminants) are being assessed [83] (J. A. Feinberg and J. Burger, unpublished data). Counterintuitively, *R. kauffeldi* persists in several locales within New York City (Staten Island) and the adjacent NJ Meadowlands. These sites are heavily industrialized and have endured severe long-term anthropogenic impacts and invasion by the common reed, *Phragmites australis*. Most offer large habitat areas, however, which may provide an important clue to survival. The surprising persistence of populations within these urban landscapes, while not completely understood, is encouraging and may have implications for management and restoration possibilities elsewhere, in the future.

We finish with a cautionary note regarding reintroductions, repatriations, and translocations. Moving species to restore extirpated populations is a common conservation and management practice, but one that can have unintended risks and consequences. For example, had a leopard frog restoration been implemented on Long Island before the 2007 discovery of extant populations on nearby Staten Island (that were later found to be *R. kauffeldi*), the incorrect species (*R. sphenocephala*) would have been moved from known populations farther to the south that harbor *R. sphenocephala*, not *R. kauffeldi*. Thus, careful consideration of systematics and population genetics at both donor and recipient site ends is critical to responsibly conducting any such endeavors.

## Conclusions

In diagnosing, describing, and defining the Atlantic Coast leopard frog, *R. kauffeldi*, we add a new and potentially at-risk cryptic vertebrate species to the northeastern and mid-Atlantic US fauna. *Rana kauffeldi* can be characterized as 1) potentially vulnerable with highly specialized and restrictive habitat needs; 2) locally abundant where present, but often only occurring in isolated and scattered locales; 3) having a restricted distribution across heavily populated, urbanized regions; and 4) having suffered extirpations from certain areas. Concerns over habitat loss and degradation continue today, along with a suite of other threats (e.g., disease, contaminants) that may pose additional future challenges.

## Supporting Information

Figure S1 **Box and whisker plots comparing the size-corrected residuals of 12 morphological characters among four** ***Rana*** **species.** Species include *R. kauffeldi* (kauf), *R. palustris* (palu), *R. pipiens* (pipi), and *R. sphenocephala* (sphe). For whisker plots, black bars  =  median, boxes  =  25^th^–75th quartiles, whiskers  =  minimum and maximum values but exclude outliers (represented by open circles). For each character, species whose measurements differed significantly (*P*<0.05) in a one-way ANOVA are denoted with different letters atop the plot. Side notches in boxes indicate significantly different medians.
(TIF)

Figure S2 **Discriminant function analyses (DFA).** Left: DFA using 12 size-corrected morphological characters measured from 264 frogs examined across four *Rana* species. Right: DFA using six bioacoustic characters measured from 45 frogs examined across five *Rana* species. Species include *R. kauffeldi* (circles), *R. sphenocephala* (triangles), *R. pipiens* (plus signs), *R. palustris* (x-crosses), and *R. sylvatica* (red squares). Morphological characters include all variables from Figure S1. Bioacoustic characters include all variables from Figure S4, except pulse rate. Black symbols twice as large in the morphological DFA represent group centroids.
(TIF)

Figure S3 **Box and whisker plots comparing spot features between** ***Rana kauffeldi*** **(kauf) and** ***R. sphenocephala*** **(sphe).** Left: total number of dorsal spots. Right: proportion of dorsal surface covered by spots. For whisker plots, black bars  =  median, boxes  = 25th–75th quartiles, whiskers  =  minimum and maximum values but exclude outliers (represented by open circles). Side notches in boxes indicate significantly different medians.
(TIF)

Figure S4 **Box and whisker plots comparing seven bioacoustic characters among five** ***Rana*** **species.** Species include *R. kauffeldi* (kauf), *R. palustris* (palu), *R. pipiens* (pipi), *R. sphenocephala* (sphe), and *R. sylvatica* (sylv). For whisker plots, black bars  =  median, boxes  = 25th–75th quartiles, whiskers  =  minimum and maximum values but exclude outliers (represented by open circles). For each character, species whose measurements differed significantly (P<0.05) in a one-way ANOVA are denoted with different letters atop the plot. Call length and call rate were temperature-corrected.
(TIF)

Table S1 **List of** ***Rana*** **specimens examined.**
(DOC)

Table S2 **List of** ***Rana*** **primary (advertisement) calls measured for bioacoustic data.**
(DOC)

Table S3 **Classification matrix for four** ***Rana*** **species using discriminant function analysis on morphometric variables.**
(DOC)

Table S4 **Coefficients for three discriminant functions (from four species of** ***Rana*** **) for each of 12 morphological characters:** head length (HL), head width (HW), eye diameter (ED), tympanum diameter (TD), foot length (FOL), eye-to-naris distance (END), naris-to-snout distance (NSD), thigh length (THL), internarial distance (IND), interorbital distance (IOD), shank length (SL), and dorsal snout angle (DSA).
(DOC)

Table S5 **Classification matrix for five** ***Rana*** **species using discriminant function analysis on bioacoustic variables.**
(DOC)

Table S6 **Coefficients for four discriminant functions (from five species of** ***Rana*** **) for each of six bioacoustic characters:** call length (CL), call rate (CR), call rise time (CRT), call duty cycle (CDC), pulse number (PN), and dominant frequency (DF).
(DOC)

Table S7 **Underlying (raw) morphometric data.**
(XLSX)

Table S8 **Underlying (raw) bioacoustic data.**
(XLSX)

Table S9 **Underlying (raw) data for color and pattern analyses.**
(XLSX)

Video S1 **A male** ***Rana kauffeldi*** **emitting its primary (advertisement) call in foreground with several other males calling in background (along with high-pitched** ***Pseudacris crucifer*** **).**
(MOV)
