# Supplementary figures and images for: Cryptic Diversity in Metropolis: Confirmation of a New Leopard Frog Species (Anura: Ranidae) from New York City and Surrounding Atlantic Coast Regions
Source: PLoS One. 2014 Oct 29;9(10):e108213. doi: 10.1371/journal.pone.0108213 (PMC4212910; doi:10.1371/journal.pone.0108213)

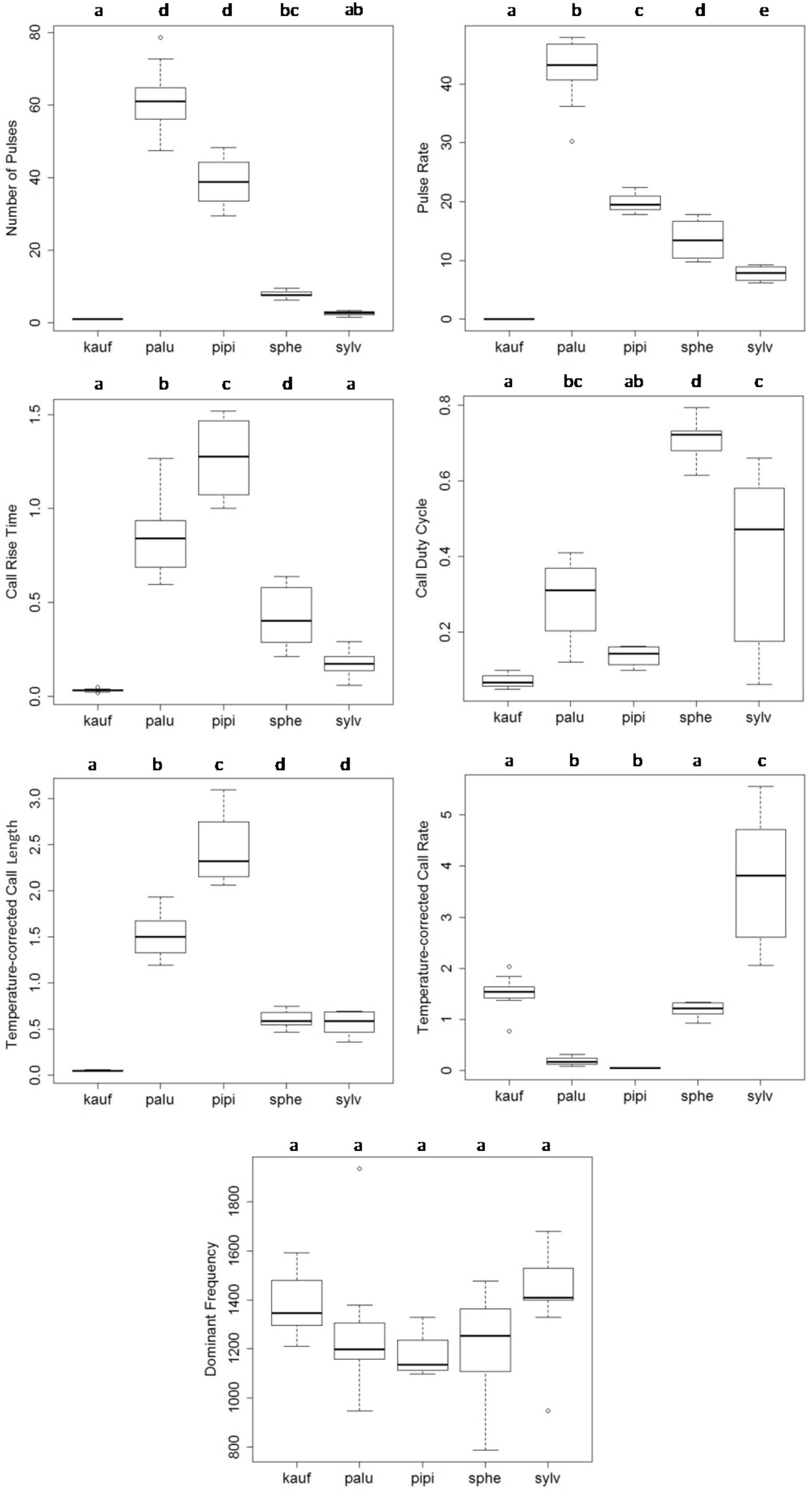

Supplement: Figure S1 — Box and whisker plots comparing the size-corrected residuals of 12 morphological characters among four Rana species. Species include R. kauffeldi (kauf), R. palustris (palu), R. pipiens (pipi), and R. sphenocephala (sphe). For whisker plots, black bars = median, boxes = 25th–75th quartiles, whiskers = minimum and maximum values but exclude outliers (represented by open circles). For each character, species whose measurements differed significantly (P<0.05) in a one-way ANOVA are denoted with different letters atop the plot. Side notches in boxes indicate significantly different medians. (TIF) [file pone.0108213.s001.tif]

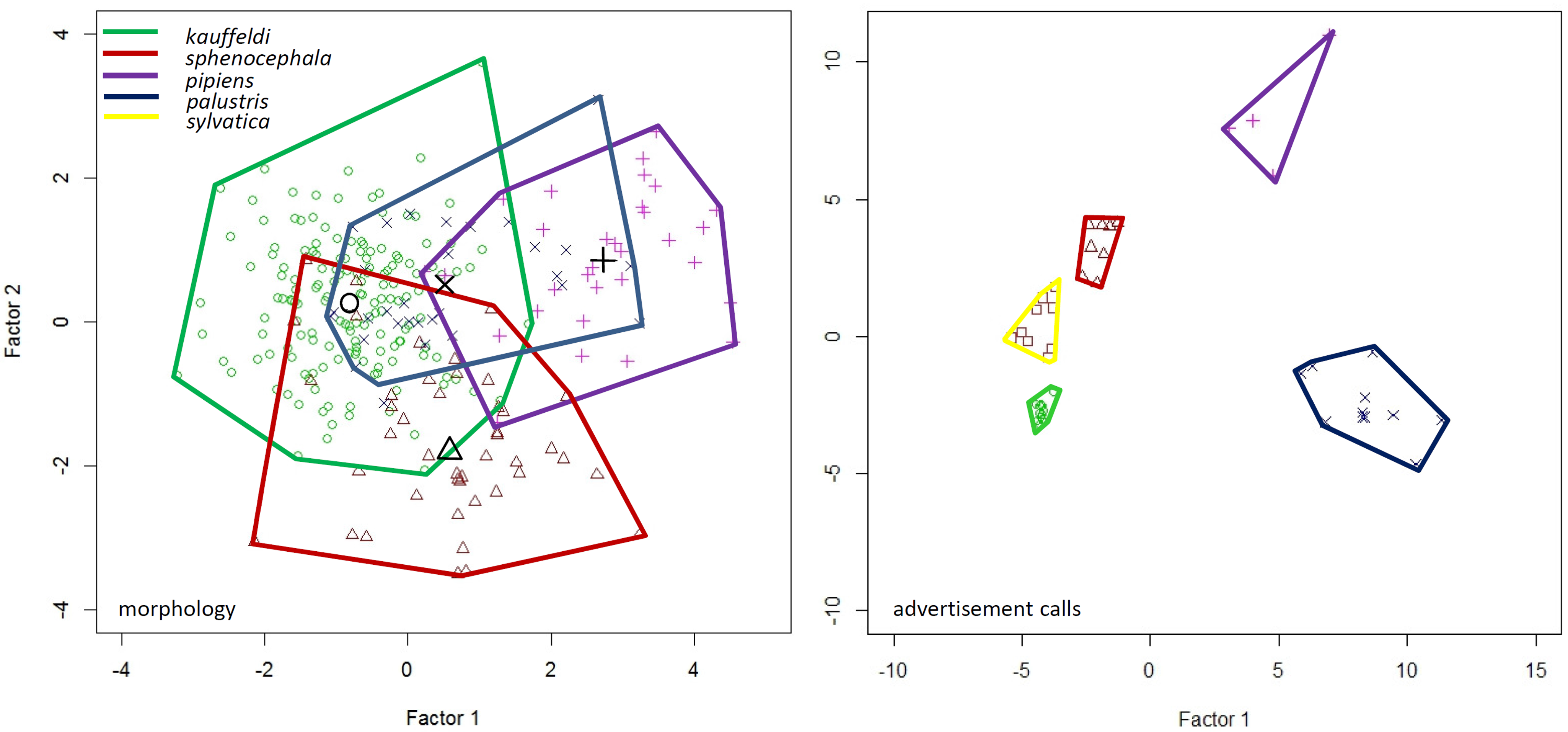

Supplement: Figure S2 — Discriminant function analyses (DFA). Left: DFA using 12 size-corrected morphological characters measured from 264 frogs examined across four Rana species. Right: DFA using six bioacoustic characters measured from 45 frogs examined across five Rana species. Species include R. kauffeldi (circles), R. sphenocephala (triangles), R. pipiens (plus signs), R. palustris (x-crosses), and R. sylvatica (red squares). Morphological characters include all variables from Figure S1. Bioacoustic characters include all variables from Figure S4, except pulse rate. Black symbols twice as large in the morphological DFA represent group centroids. (TIF) [file pone.0108213.s002.tif]

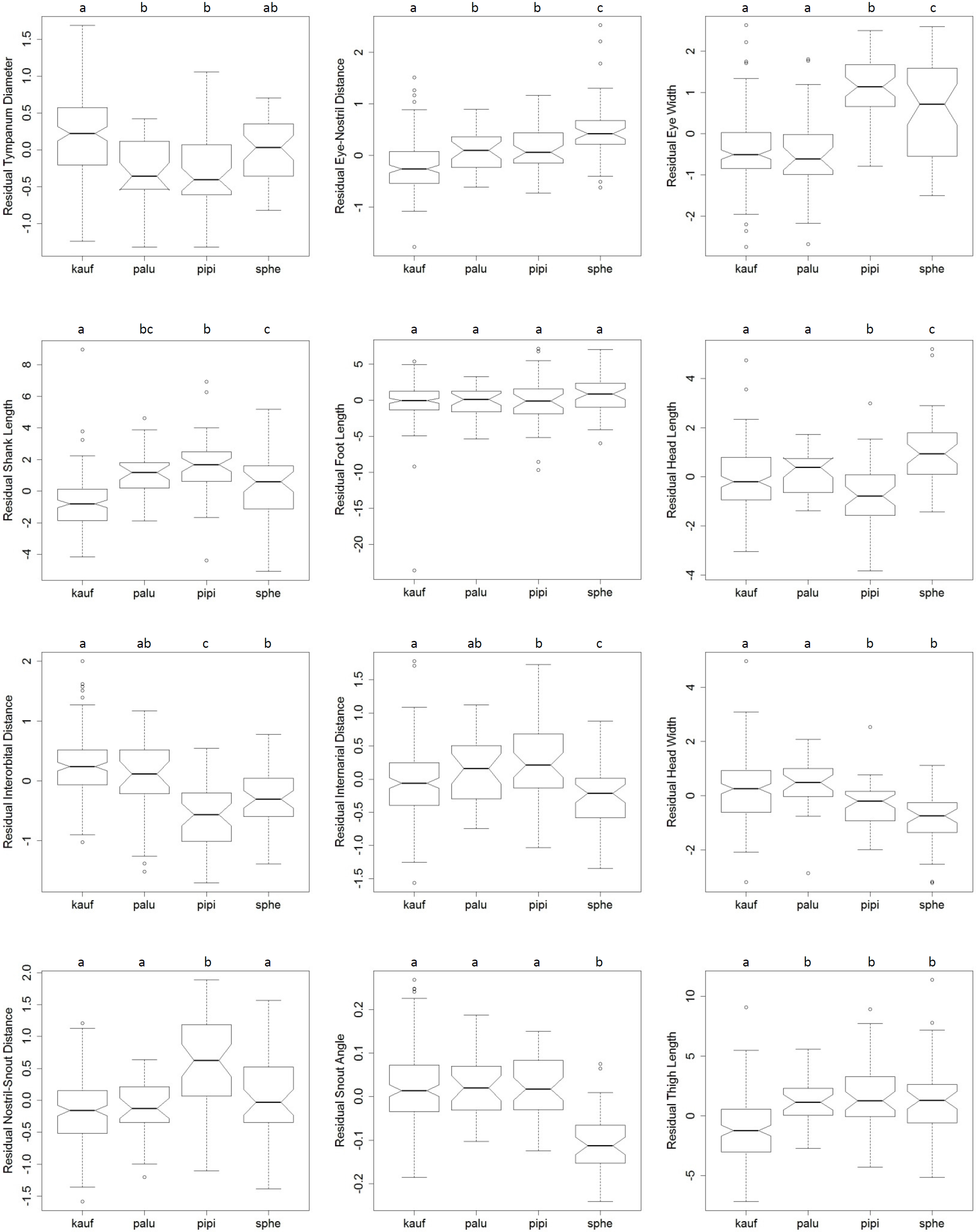

Supplement: Figure S3 — Box and whisker plots comparing spot features between Rana kauffeldi (kauf) and R. sphenocephala (sphe). Left: total number of dorsal spots. Right: proportion of dorsal surface covered by spots. For whisker plots, black bars = median, boxes = 25th–75th quartiles, whiskers = minimum and maximum values but exclude outliers (represented by open circles). Side notches in boxes indicate significantly different medians. (TIF) [file pone.0108213.s003.tif]

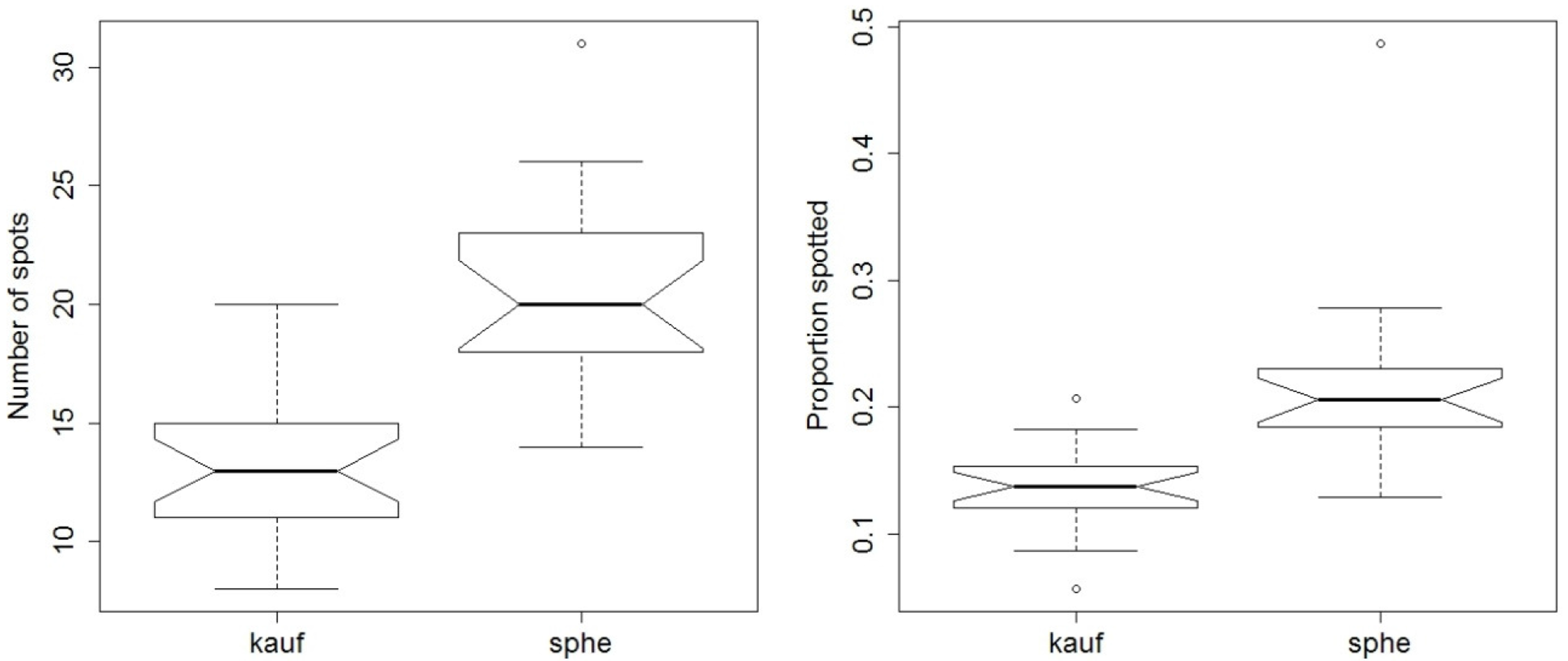

Supplement: Figure S4 — Box and whisker plots comparing seven bioacoustic characters among five Rana species. Species include R. kauffeldi (kauf), R. palustris (palu), R. pipiens (pipi), R. sphenocephala (sphe), and R. sylvatica (sylv). For whisker plots, black bars = median, boxes = 25th–75th quartiles, whiskers = minimum and maximum values but exclude outliers (represented by open circles). For each character, species whose measurements differed significantly (P<0.05) in a one-way ANOVA are denoted with different letters atop the plot. Call length and call rate were temperature-corrected. (TIF) [file pone.0108213.s004.tif]
