# Supplementary material for: Cryptic Diversity in Metropolis: Confirmation of a New Leopard Frog Species (Anura: Ranidae) from New York City and Surrounding Atlantic Coast Regions
Source: PLoS One. 2014 Oct 29;9(10):e108213. doi: 10.1371/journal.pone.0108213 (PMC4212910; doi:10.1371/journal.pone.0108213)
Supplement: Table S1 — List of Rana specimens examined. (DOC) [file pone.0108213.s005.doc]

| **Table S1.** List of *Rana* specimens examined. Fluid specimens were examined from the following museums: Yale Peabody Museum (YPM), American Museum of Natural History (AMNH), Academy of Natural Sciences of Drexel University (ANSP), Carnegie Museum of Natural History (CM), and Sam Noble Oklahoma Museum (OMNH). Analysis type codes are as follows: morphometric analysis (M), dorsal spot analyses (number of spots and percent coverage) (D), dorsal spot shape analysis (SH), snout spot analysis (SN), color analysis (C), and femur reticulum analysis (F). Two additional codes indicate ‘examination-only’, for femur reticulum (R) and extirpation zone composition (E); these were not used in any specific analysis. * = Foot length unavailable, not included in morphometric analysis. † = Photo voucher only, deposited at YPM. †† = Photo voucher with tissue, deposited at YPM. § = Single photo voucher of amplexed pair. §§ = Holotype. | | | | | | | |
| --- | --- | --- | --- | --- | --- | --- | --- |
| Taxon | Museum | Specimen No. | Notes | State/Province | County/Region | Analysis Type |  |
| *R. kauffeldi* | YPM | 13559 |  | CT | Middlesex | M | * |
| *R. kauffeldi* | YPM | 13560 |  | CT | Middlesex | M | * |
| *R. kauffeldi* | YPM | 13561 |  | CT | Middlesex | M | * |
| *R. kauffeldi* | AMNH | 35503 | see Moore (1944) Pl. 62, #5 | NJ | Bergen | D,M,SN |  |
| *R. kauffeldi* | AMNH | 35512 | see Moore (1944) Pl. 62, #6 | NJ | Bergen | D,M,SN |  |
| *R. kauffeldi* | AMNH | 84220 |  | NJ | Bergen | F |  |
| *R. kauffeldi* | AMNH | 121324 |  | NJ | Bergen | F |  |
| *R. kauffeldi* | AMNH | 121340 |  | NJ | Bergen | F |  |
| *R. kauffeldi* | AMNH | 121341 |  | NJ | Bergen | F |  |
| *R. kauffeldi* | AMNH | 1720 |  | NJ | Bergen | M |  |
| *R. kauffeldi* | AMNH | 1721 |  | NJ | Bergen | M |  |
| *R. kauffeldi* | AMNH | 12855 |  | NJ | Bergen | M |  |
| *R. kauffeldi* | AMNH | 35504 |  | NJ | Bergen | M |  |
| *R. kauffeldi* | AMNH | 35505 |  | NJ | Bergen | M |  |
| *R. kauffeldi* | AMNH | 35509 |  | NJ | Bergen | M |  |
| *R. kauffeldi* | AMNH | 35510 |  | NJ | Bergen | M |  |
| *R. kauffeldi* | AMNH | 35511 |  | NJ | Bergen | M |  |
| *R. kauffeldi* | AMNH | 52344 |  | NJ | Bergen | M |  |
| *R. kauffeldi* | AMNH | 63848 |  | NJ | Bergen | M |  |
| *R. kauffeldi* | AMNH | 63849 |  | NJ | Bergen | M |  |
| *R. kauffeldi* | AMNH | 63850 |  | NJ | Bergen | M |  |
| *R. kauffeldi* | AMNH | 67491 |  | NJ | Bergen | M |  |
| *R. kauffeldi* | AMNH | 81337 |  | NJ | Bergen | M |  |
| *R. kauffeldi* | AMNH | 81338 |  | NJ | Bergen | M |  |
| *R. kauffeldi* | AMNH | 81339 |  | NJ | Bergen | M |  |
| *R. kauffeldi* | AMNH | 81840 |  | NJ | Bergen | M |  |
| *R. kauffeldi* | AMNH | 121124 |  | NJ | Bergen | M |  |
| **Table S1.** | **Continued** |  |  |  |  |  |  |
| Taxon | Museum | Specimen No. | Notes | State/Province | County/Region | Analysis Type |  |
| *R. kauffeldi* | AMNH | 121190 |  | NJ | Essex | M |  |
| *R. kauffeldi* | AMNH | 121191 |  | NJ | Essex | M |  |
| *R. kauffeldi* | AMNH | 121192 |  | NJ | Essex | M |  |
| *R. kauffeldi* | AMNH | 121193 |  | NJ | Essex | M |  |
| *R. kauffeldi* | AMNH | 121194 |  | NJ | Essex | M |  |
| *R. kauffeldi* | AMNH | 121195 |  | NJ | Essex | M |  |
| *R. kauffeldi* | AMNH | 121198 |  | NJ | Essex | M |  |
| *R. kauffeldi* | AMNH | 121199 |  | NJ | Essex | M |  |
| *R. kauffeldi* | AMNH | 121200 |  | NJ | Essex | M |  |
| *R. kauffeldi* | AMNH | 121203 |  | NJ | Essex | M |  |
| *R. kauffeldi* | AMNH | 121204 |  | NJ | Essex | M |  |
| *R. kauffeldi* | AMNH | 121205 |  | NJ | Essex | M |  |
| *R. kauffeldi* | AMNH | 121206 |  | NJ | Essex | M |  |
| *R. kauffeldi* | AMNH | 121208 |  | NJ | Essex | M |  |
| *R. kauffeldi* | AMNH | 121209 |  | NJ | Essex | M |  |
| *R. kauffeldi* | AMNH | 121210 |  | NJ | Essex | M |  |
| *R. kauffeldi* | AMNH | 121212 |  | NJ | Essex | M |  |
| *R. kauffeldi* | AMNH | 121213 |  | NJ | Essex | M |  |
| *R. kauffeldi* | AMNH | 121214 |  | NJ | Essex | M |  |
| *R. kauffeldi* | AMNH | 121215 |  | NJ | Essex | M |  |
| *R. kauffeldi* | AMNH | 121216 |  | NJ | Essex | M |  |
| *R. kauffeldi* | AMNH | 121217 |  | NJ | Essex | M |  |
| *R. kauffeldi* | AMNH | 121218 |  | NJ | Essex | M |  |
| *R. kauffeldi* | AMNH | 121219 |  | NJ | Essex | M |  |
| *R. kauffeldi* | AMNH | 121220 |  | NJ | Essex | M |  |
| *R. kauffeldi* | AMNH | 121221 |  | NJ | Essex | M |  |
| *R. kauffeldi* | AMNH | 121222 |  | NJ | Essex | M |  |
| *R. kauffeldi* | AMNH | 121223 |  | NJ | Essex | M |  |
| *R. kauffeldi* | AMNH | 121224 |  | NJ | Essex | M |  |
| *R. kauffeldi* | AMNH | 121225 |  | NJ | Essex | M |  |
| *R. kauffeldi* | AMNH | 121226 |  | NJ | Essex | M |  |
| *R. kauffeldi* | AMNH | 121227 |  | NJ | Essex | M |  |
| *R. kauffeldi* | AMNH | 121228 |  | NJ | Essex | M |  |
| **Table S1.** | **Continued** |  |  |  |  |  |  |
| Taxon | Museum | Specimen No. | Notes | State/Province | County/Region | Analysis Type |  |
| *R. kauffeldi* | AMNH | 121229 |  | NJ | Essex | M |  |
| *R. kauffeldi* | AMNH | 121230 |  | NJ | Essex | M |  |
| *R. kauffeldi* | AMNH | 121231 |  | NJ | Essex | M |  |
| *R. kauffeldi* | AMNH | 121232 |  | NJ | Essex | M |  |
| *R. kauffeldi* | AMNH | 121233 |  | NJ | Essex | M |  |
| *R. kauffeldi* | AMNH | 121234 |  | NJ | Essex | M |  |
| *R. kauffeldi* | AMNH | 121235 |  | NJ | Essex | M |  |
| *R. kauffeldi* | AMNH | 121236 |  | NJ | Essex | M |  |
| *R. kauffeldi* | AMNH | 121237 |  | NJ | Essex | M |  |
| *R. kauffeldi* | AMNH | 121238 |  | NJ | Essex | M |  |
| *R. kauffeldi* | AMNH | 121240 |  | NJ | Essex | M |  |
| *R. kauffeldi* | AMNH | 121241 |  | NJ | Essex | M |  |
| *R. kauffeldi* | AMNH | 121243 |  | NJ | Essex | M |  |
| *R. kauffeldi* | AMNH | 121244 |  | NJ | Essex | M |  |
| *R. kauffeldi* | AMNH | 121245 |  | NJ | Essex | M |  |
| *R. kauffeldi* | AMNH | 51018 |  | NJ | Morris | M |  |
| *R. kauffeldi* | AMNH | 51019 |  | NJ | Morris | M |  |
| *R. kauffeldi* | AMNH | 51020 |  | NJ | Morris | M |  |
| *R. kauffeldi* | AMNH | 51021 |  | NJ | Morris | M |  |
| *R. kauffeldi* | AMNH | 51022 |  | NJ | Morris | M |  |
| *R. kauffeldi* | AMNH | 64499 |  | NJ | Morris | M |  |
| *R. kauffeldi* | AMNH | 79579 |  | NJ | Morris | M |  |
| *R. kauffeldi* | AMNH | 79580 |  | NJ | Morris | M |  |
| *R. kauffeldi* | AMNH | 79581 |  | NJ | Morris | M |  |
| *R. kauffeldi* | AMNH | 84181 |  | NJ | Morris | M |  |
| *R. kauffeldi* | AMNH | 121321 |  | NJ | Morris | M |  |
| *R. kauffeldi* | AMNH | 121322 |  | NJ | Morris | M |  |
| *R. kauffeldi* | AMNH | 121323 |  | NJ | Morris | M |  |
| *R. kauffeldi* | AMNH | 121325 |  | NJ | Morris | M |  |
| *R. kauffeldi* | AMNH | 121326 |  | NJ | Morris | M |  |
| *R. kauffeldi* | AMNH | 121327 |  | NJ | Morris | M |  |
| *R. kauffeldi* | AMNH | 121328 |  | NJ | Morris | M |  |
| *R. kauffeldi* | AMNH | 121329 |  | NJ | Morris | M |  |
| **Table S1.** | **Continued** |  |  |  |  |  |  |
| Taxon | Museum | Specimen No. | Notes | State/Province | County/Region | Analysis Type |  |
| *R. kauffeldi* | AMNH | 121330 |  | NJ | Morris | M |  |
| *R. kauffeldi* | AMNH | 121331 |  | NJ | Morris | M |  |
| *R. kauffeldi* | AMNH | 121332 |  | NJ | Morris | M |  |
| *R. kauffeldi* | AMNH | 121333 |  | NJ | Morris | M |  |
| *R. kauffeldi* | AMNH | 121334 |  | NJ | Morris | M |  |
| *R. kauffeldi* | AMNH | 121335 |  | NJ | Morris | M |  |
| *R. kauffeldi* | AMNH | 121336 |  | NJ | Morris | M |  |
| *R. kauffeldi* | AMNH | 121337 |  | NJ | Morris | M |  |
| *R. kauffeldi* | AMNH | 121338 |  | NJ | Morris | M |  |
| *R. kauffeldi* | AMNH | 121339 |  | NJ | Morris | M |  |
| *R. kauffeldi* | AMNH | 121342 |  | NJ | Morris | M |  |
| *R. kauffeldi* | AMNH | 121343 |  | NJ | Morris | M |  |
| *R. kauffeldi* | AMNH | 121344 |  | NJ | Morris | M |  |
| *R. kauffeldi* | AMNH | 18730 |  | NJ | Passaic | M |  |
| *R. kauffeldi* | AMNH | 70296 |  | NJ | Passaic | M |  |
| *R. kauffeldi* | AMNH | 70366 |  | NJ | Passaic | M |  |
| *R. kauffeldi* | AMNH | 70367 |  | NJ | Passaic | M |  |
| *R. kauffeldi* | AMNH | 77415 |  | NJ | Passaic | M |  |
| *R. kauffeldi* | AMNH | 79391 |  | NJ | Passaic | M |  |
| *R. kauffeldi* | AMNH | 121103 |  | NJ | Passaic | M |  |
| *R. kauffeldi* | AMNH | 121104 |  | NJ | Passaic | M |  |
| *R. kauffeldi* | AMNH | 121105 |  | NJ | Passaic | M |  |
| *R. kauffeldi* | AMNH | 121106 |  | NJ | Passaic | M |  |
| *R. kauffeldi* | AMNH | 121107 |  | NJ | Passaic | M |  |
| *R. kauffeldi* | AMNH | 121113 |  | NJ | Somerset | M |  |
| *R. kauffeldi* | AMNH | 121114 |  | NJ | Somerset | M |  |
| *R. kauffeldi* | AMNH | 121116 |  | NJ | Somerset | M |  |
| *R. kauffeldi* | AMNH | 121117 |  | NJ | Somerset | M |  |
| *R. kauffeldi* | AMNH | 121118 |  | NJ | Somerset | M |  |
| *R. kauffeldi* | AMNH | 121119 |  | NJ | Somerset | M |  |
| *R. kauffeldi* | AMNH | 121120 |  | NJ | Somerset | M |  |
| *R. kauffeldi* | AMNH | 121121 |  | NJ | Somerset | M |  |
| *R. kauffeldi* | AMNH | 121316 |  | NJ | Union | M |  |
| **Table S1.** | **Continued** |  |  |  |  |  |  |
| Taxon | Museum | Specimen No. | Notes | State/Province | County/Region | Analysis Type |  |
| *R. kauffeldi* | AMNH | 121317 |  | NJ | Union | M |  |
| *R. kauffeldi* | AMNH | 121318 |  | NJ | Union | M |  |
| *R. kauffeldi* | AMNH | 121319 |  | NJ | Union | M |  |
| *R. kauffeldi* | AMNH | 121320 |  | NJ | Union | M |  |
| *R. kauffeldi* | AMNH | 52342 |  | NY | Bronx | E,M |  |
| *R. kauffeldi* | AMNH | 106551 |  | NY | Bronx | E,M |  |
| *R. kauffeldi* | AMNH | 106552 |  | NY | Bronx | E,M |  |
| *R. kauffeldi* | AMNH | 106553 |  | NY | Bronx | E,M |  |
| *R. kauffeldi* | AMNH | 106554 |  | NY | Bronx | E,M |  |
| *R. kauffeldi* | AMNH | 41292 | Long Island | NY | Nassau | E |  |
| *R. kauffeldi* | AMNH | 21016 |  | NY | Orange | M |  |
| *R. kauffeldi* | AMNH | 103134 |  | NY | Orange | M |  |
| *R. kauffeldi* | AMNH | 103135 |  | NY | Orange | M |  |
| *R. kauffeldi* | AMNH | 164382 |  | NY | Orange | M |  |
| *R. kauffeldi* | AMNH | 14386 | Long Island | NY | Queens | E |  |
| *R. kauffeldi* | AMNH | 14515 | Long Island | NY | Queens | E |  |
| *R. kauffeldi* | AMNH | 14516 | Long Island | NY | Queens | E |  |
| *R. kauffeldi* | AMNH | 14517 | Long Island | NY | Queens | E |  |
| *R. kauffeldi* | AMNH | 14518 | Long Island | NY | Queens | E |  |
| *R. kauffeldi* | AMNH | 14519 | Long Island | NY | Queens | E |  |
| *R. kauffeldi* | AMNH | 14520 | Long Island | NY | Queens | E |  |
| *R. kauffeldi* | AMNH | 14521 | Long Island | NY | Queens | E |  |
| *R. kauffeldi* | AMNH | 14522 | Long Island | NY | Queens | E |  |
| *R. kauffeldi* | AMNH | 38193 | Long Island | NY | Queens | E |  |
| *R. kauffeldi* | AMNH | 38194 | Long Island | NY | Queens | E |  |
| *R. kauffeldi* | AMNH | 38195 | Long Island | NY | Queens | E |  |
| *R. kauffeldi* | AMNH | 23030 | see Moore (1944) Pl. 62, #4 | NY | Richmond | D,M,SN |  |
| *R. kauffeldi* | AMNH | 121857 |  | NY | Richmond | F,M |  |
| *R. kauffeldi* | AMNH | 121858 |  | NY | Richmond | F,M |  |
| *R. kauffeldi* | AMNH | 581 |  | NY | Richmond | M |  |
| *R. kauffeldi* | AMNH | 636 |  | NY | Richmond | M |  |
| *R. kauffeldi* | AMNH | 3542 |  | NY | Richmond | M |  |
| *R. kauffeldi* | AMNH | 3543 |  | NY | Richmond | M |  |
| **Table S1.** | **Continued** |  |  |  |  |  |  |
| Taxon | Museum | Specimen No. | Notes | State/Province | County/Region | Analysis Type |  |
| *R. kauffeldi* | AMNH | 3699 |  | NY | Richmond | M |  |
| *R. kauffeldi* | AMNH | 3700 |  | NY | Richmond | M |  |
| *R. kauffeldi* | AMNH | 23029 |  | NY | Richmond | M |  |
| *R. kauffeldi* | AMNH | 23031 |  | NY | Richmond | M |  |
| *R. kauffeldi* | AMNH | 23032 |  | NY | Richmond | M |  |
| *R. kauffeldi* | AMNH | 125959 |  | NY | Richmond | M |  |
| *R. kauffeldi* | AMNH | 125960 |  | NY | Richmond | M |  |
| *R. kauffeldi* | AMNH | 125961 |  | NY | Richmond | M |  |
| *R. kauffeldi* | AMNH | 125962 |  | NY | Richmond | M |  |
| *R. kauffeldi* | YPM | 13791 |  | NY | Richmond | C | † |
| *R. kauffeldi* | YPM | 13822 |  | NY | Richmond | C | † |
| *R. kauffeldi* | YPM | 13823 |  | NY | Richmond | C | † |
| *R. kauffeldi* | YPM | 13828 |  | NY | Richmond | C | † |
| *R. kauffeldi* | YPM | 13832 |  | NY | Richmond | C | † |
| *R. kauffeldi* | YPM | 13833 |  | NY | Richmond | C | † |
| *R. kauffeldi* | YPM | 13837 |  | NY | Richmond | C | † |
| *R. kauffeldi* | YPM | 13843 |  | NY | Richmond | C | † |
| *R. kauffeldi* | YPM | 13844 |  | NY | Richmond | C | † |
| *R. kauffeldi* | YPM | 13845 |  | NY | Richmond | C | †† |
| *R. kauffeldi* | YPM | 14018 |  | NY | Richmond | C | † |
| *R. kauffeldi* | YPM | 13217 |  | NY | Richmond | C,D,F,M,SH,SN | §§ |
| *R. kauffeldi* | YPM | 13788 |  | NY | Richmond | C,D,F,SH,SN | † |
| *R. kauffeldi* | YPM | 13789 |  | NY | Richmond | C,D,F,SH,SN | † |
| *R. kauffeldi* | YPM | 13847 |  | NY | Richmond | C,D,F,SH,SN | † |
| *R. kauffeldi* | YPM | 13851 | Top frog | NY | Richmond | C,D,F,SH,SN | † |
| *R. kauffeldi* | YPM | 13853 |  | NY | Richmond | C,D,F,SH,SN | † |
| *R. kauffeldi* | YPM | 13854 |  | NY | Richmond | C,D,F,SH,SN | † |
| *R. kauffeldi* | YPM | 14020 |  | NY | Richmond | C,D,F,SH,SN | † |
| *R. kauffeldi* | YPM | 14021 |  | NY | Richmond | C,D,F,SH,SN | † |
| *R. kauffeldi* | YPM | 14107 |  | NY | Richmond | C,D,F,SH,SN | †† |
| *R. kauffeldi* | YPM | 13820 |  | NY | Richmond | C,D,SH,SN | † |
| *R. kauffeldi* | YPM | 13821 |  | NY | Richmond | C,D,SH,SN | † |
| *R. kauffeldi* | YPM | 14022 |  | NY | Richmond | C,D,SH,SN | † |
| **Table S1.** | **Continued** |  |  |  |  |  |  |
| Taxon | Museum | Specimen No. | Notes | State/Province | County/Region | Analysis Type |  |
| *R. kauffeldi* | YPM | 14027 |  | NY | Richmond | C,D,SH,SN | † |
| *R. kauffeldi* | YPM | 14029 |  | NY | Richmond | C,D,SH,SN | † |
| *R. kauffeldi* | YPM | 14108 |  | NY | Richmond | C,D,SH,SN | †† |
| *R. kauffeldi* | YPM | 13866 |  | NY | Richmond | C,D,SN | † |
| *R. kauffeldi* | YPM | 13862 |  | NY | Richmond | C,F,SH,SN | † |
| *R. kauffeldi* | YPM | 13863 |  | NY | Richmond | C,F,SH,SN | † |
| *R. kauffeldi* | YPM | 13865 |  | NY | Richmond | C,F,SH,SN | † |
| *R. kauffeldi* | YPM | 13920 |  | NY | Richmond | C,F,SH,SN |  |
| *R. kauffeldi* | YPM | 13921 |  | NY | Richmond | C,F,SH,SN |  |
| *R. kauffeldi* | YPM | 14024 |  | NY | Richmond | C,F,SH,SN | † |
| *R. kauffeldi* | YPM | 14025 |  | NY | Richmond | C,F,SH,SN | † |
| *R. kauffeldi* | YPM | 14111 |  | NY | Richmond | C,F,SN | † |
| *R. kauffeldi* | YPM | 13790 |  | NY | Richmond | C,SH,SN | † |
| *R. kauffeldi* | YPM | 13827 |  | NY | Richmond | C,SH,SN | †† |
| *R. kauffeldi* | YPM | 13836 |  | NY | Richmond | C,SH,SN | † |
| *R. kauffeldi* | YPM | 13838 |  | NY | Richmond | C,SH,SN | † |
| *R. kauffeldi* | YPM | 13840 |  | NY | Richmond | C,SH,SN | †† |
| *R. kauffeldi* | YPM | 13842 |  | NY | Richmond | C,SH,SN | †† |
| *R. kauffeldi* | YPM | 13848 |  | NY | Richmond | C,SH,SN | † |
| *R. kauffeldi* | YPM | 13849 |  | NY | Richmond | C,SH,SN | † |
| *R. kauffeldi* | YPM | 13864 | Amplexed male | NY | Richmond | C,SH,SN | § |
| *R. kauffeldi* | YPM | 13872 |  | NY | Richmond | C,SH,SN | † |
| *R. kauffeldi* | YPM | 14019 |  | NY | Richmond | C,SH,SN | † |
| *R. kauffeldi* | YPM | 14023 |  | NY | Richmond | C,SH,SN | † |
| *R. kauffeldi* | YPM | 14026 |  | NY | Richmond | C,SH,SN | † |
| *R. kauffeldi* | YPM | 14028 |  | NY | Richmond | C,SH,SN | † |
| *R. kauffeldi* | YPM | 14048 |  | NY | Richmond | C,SH,SN | † |
| *R. kauffeldi* | YPM | 14086 |  | NY | Richmond | C,SH,SN | † |
| *R. kauffeldi* | YPM | 14110 |  | NY | Richmond | C,SH,SN | † |
| *R. kauffeldi* | YPM | 13768 | Amplexed female | NY | Richmond | C,SN | § |
| *R. kauffeldi* | YPM | 13768 | Amplexed male | NY | Richmond | C,SN | § |
| *R. kauffeldi* | YPM | 13792 |  | NY | Richmond | C,SN | † |
| *R. kauffeldi* | YPM | 13793 | Bottom frog | NY | Richmond | C,SN | † |
| **Table S1.** | **Continued** |  |  |  |  |  |  |
| Taxon | Museum | Specimen No. | Notes | State/Province | County/Region | Analysis Type |  |
| *R. kauffeldi* | YPM | 13824 |  | NY | Richmond | C,SN | † |
| *R. kauffeldi* | YPM | 13825 |  | NY | Richmond | C,SN | † |
| *R. kauffeldi* | YPM | 13826 |  | NY | Richmond | C,SN | † |
| *R. kauffeldi* | YPM | 13829 |  | NY | Richmond | C,SN | †† |
| *R. kauffeldi* | YPM | 13830 |  | NY | Richmond | C,SN | † |
| *R. kauffeldi* | YPM | 13831 |  | NY | Richmond | C,SN | † |
| *R. kauffeldi* | YPM | 13834 |  | NY | Richmond | C,SN | † |
| *R. kauffeldi* | YPM | 13835 |  | NY | Richmond | C,SN | † |
| *R. kauffeldi* | YPM | 13841 |  | NY | Richmond | C,SN | † |
| *R. kauffeldi* | YPM | 13864 | Amplexed female | NY | Richmond | C,SN | § |
| *R. kauffeldi* | YPM | 13867 |  | NY | Richmond | C,SN | † |
| *R. kauffeldi* | YPM | 13868 |  | NY | Richmond | C,SN | † |
| *R. kauffeldi* | YPM | 13869 |  | NY | Richmond | C,SN | † |
| *R. kauffeldi* | YPM | 13870 |  | NY | Richmond | C,SN | † |
| *R. kauffeldi* | YPM | 13871 |  | NY | Richmond | C,SN | † |
| *R. kauffeldi* | YPM | 14017 |  | NY | Richmond | C,SN | † |
| *R. kauffeldi* | YPM | 14032 |  | NY | Richmond | C,SN | † |
| *R. kauffeldi* | YPM | 14033 |  | NY | Richmond | C,SN | † |
| *R. kauffeldi* | YPM | 13850 |  | NY | Richmond | D,F,SH,SN | † |
| *R. kauffeldi* | YPM | 13767 |  | NY | Richmond | F | † |
| *R. kauffeldi* | YPM | 14112 |  | NY | Richmond | F,SH,SN |  |
| *R. kauffeldi* | AMNH | 5369 | Long Island | NY | Suffolk | E |  |
| *R. kauffeldi* | AMNH | 18674 | Long Island | NY | Suffolk | E |  |
| *R. kauffeldi* | AMNH | 121856 | Long Island | NY | Suffolk | E |  |
| *R. kauffeldi* | AMNH | 125957 | Long Island | NY | Suffolk | E |  |
| *R. kauffeldi* | AMNH | 125958 | Long Island | NY | Suffolk | E |  |
| *R. kauffeldi* | AMNH | 130217 | Long Island | NY | Suffolk | E |  |
| *R. kauffeldi* | AMNH | 130218 | Long Island | NY | Suffolk | E |  |
| *R. kauffeldi* | AMNH | 132936 | Long Island | NY | Suffolk | E |  |
| *R. kauffeldi* | Overton (1941a) | Figure 34 | Long Island | NY | Suffolk | E |  |
| *R. kauffeldi* | Overton (1941a) | Figure 38 | Long Island | NY | Suffolk | E |  |
| *R. kauffeldi* | Overton (1941b) | Page 34 (right) | Long Island | NY | Suffolk | E |  |
| *R. kauffeldi* | ANSP | 17675 |  | PA | Bucks | M |  |
| **Table S1.** | **Continued** |  |  |  |  |  |  |
| Taxon | Museum | Specimen No. | Notes | State/Province | County/Region | Analysis Type |  |
| *R. kauffeldi* | ANSP | 32684 |  | PA | Bucks | M |  |
| *R. kauffeldi* | ANSP | 2787 |  | PA | Philadelphia | M |  |
| *R. kauffeldi* | ANSP | 2788 |  | PA | Philadelphia | M |  |
| *R. kauffeldi* | ANSP | 2804 |  | PA | Philadelphia | M |  |
| *R. kauffeldi* | ANSP | 2879 |  | PA | Philadelphia | M |  |
| *R. kauffeldi* | ANSP | 2892 |  | PA | Philadelphia | M |  |
| *R. kauffeldi* | ANSP | 19222 |  | PA | Philadelphia | M |  |
| *R. kauffeldi* | Porter (1941) | Figure 1 | Right photo | PA | Philadelphia | D,SN |  |
| *R. palustris* | YPM | 6103 |  | CT | Fairfield | M |  |
| *R. palustris* | YPM | 7142 |  | CT | Fairfield | M |  |
| *R. palustris* | YPM | 10631 |  | CT | Fairfield | M |  |
| *R. palustris* | YPM | 10303 |  | CT | Hartford | M |  |
| *R. palustris* | YPM | 10304 |  | CT | Hartford | M |  |
| *R. palustris* | YPM | 10307 |  | CT | Hartford | M |  |
| *R. palustris* | YPM | 10308 |  | CT | Hartford | M |  |
| *R. palustris* | YPM | 11757 |  | CT | Hartford | M |  |
| *R. palustris* | YPM | 9018 |  | CT | Litchfield | M |  |
| *R. palustris* | YPM | 9110 |  | CT | Litchfield | M |  |
| *R. palustris* | YPM | 1070 |  | CT | New Haven | M |  |
| *R. palustris* | YPM | 1074 |  | CT | New Haven | M |  |
| *R. palustris* | YPM | 2974 |  | CT | New Haven | M |  |
| *R. palustris* | YPM | 3067 |  | CT | New Haven | M |  |
| *R. palustris* | YPM | 3129 |  | CT | New Haven | M |  |
| *R. palustris* | YPM | 3133 |  | CT | New Haven | M |  |
| *R. palustris* | YPM | 3134 |  | CT | New Haven | M |  |
| *R. palustris* | YPM | 3190 |  | CT | New Haven | M |  |
| *R. palustris* | YPM | 4907 |  | CT | New Haven | M |  |
| *R. palustris* | YPM | 4908 |  | CT | New Haven | M |  |
| *R. palustris* | YPM | 4909 |  | CT | New Haven | M |  |
| *R. palustris* | YPM | 10225 |  | CT | New Haven | M |  |
| *R. palustris* | YPM | 10684 |  | CT | New Haven | M |  |
| *R. palustris* | YPM | 6673 |  | CT | New London | M |  |
| *R. palustris* | YPM | 6647 |  | CT | Tolland | M |  |
| **Table S1.** | **Continued** |  |  |  |  |  |  |
| Taxon | Museum | Specimen No. | Notes | State/Province | County/Region | Analysis Type |  |
| *R. palustris* | YPM | 10266 |  | CT | Tolland | M |  |
| *R. palustris* | YPM | 12681 |  | ME | Franklin | M |  |
| *R. palustris* | YPM | 6357 |  | ME | Hancock | M |  |
| *R. palustris* | YPM | 12520 |  | ME | Somerset | M |  |
| *R. palustris* | YPM | 12680 |  | ME | Somerset | M |  |
| *R. pipiens* | YPM | 9315 |  | CT | Litchfield | F,M |  |
| *R. pipiens* | YPM | 13052 |  | CT | Litchfield | F,M |  |
| *R. pipiens* | YPM | 13564 |  | CT | Litchfield | F,M | * |
| *R. pipiens* | YPM | 13565 |  | CT | Litchfield | F,M | * |
| *R. pipiens* | YPM | 13566 |  | CT | Litchfield | F,M | * |
| *R. pipiens* | YPM | 13567 |  | CT | Litchfield | F,M | * |
| *R. pipiens* | YPM | 13568 |  | CT | Litchfield | F,M | * |
| *R. pipiens* | YPM | 13569 |  | CT | Litchfield | F,M |  |
| *R. pipiens* | YPM | 13570 |  | CT | Litchfield | F,M | * |
| *R. pipiens* | YPM | 13571 |  | CT | Litchfield | F,M | * |
| *R. pipiens* | YPM | 13572 |  | CT | Litchfield | F,M | * |
| *R. pipiens* | YPM | 13573 |  | CT | Litchfield | F,M | * |
| *R. pipiens* | YPM | 13574 |  | CT | Litchfield | F,M | * |
| *R. pipiens* | YPM | 13575 |  | CT | Litchfield | F,M | * |
| *R. pipiens* | YPM | 13576 |  | CT | Litchfield | F,M | * |
| *R. pipiens* | YPM | 13577 |  | CT | Litchfield | F,M | * |
| *R. pipiens* | YPM | 13578 |  | CT | Litchfield | F,M | * |
| *R. pipiens* | YPM | 13612 |  | CT | Litchfield | F,M |  |
| *R. pipiens* | YPM | 13562 |  | CT | Middlesex | F,M | * |
| *R. pipiens* | YPM | 13563 |  | CT | Middlesex | M | * |
| *R. pipiens* | YPM | 1022 |  | CT | New Haven | E,F,M |  |
| *R. pipiens* | YPM | 1077 |  | CT | New Haven | E,F,M |  |
| *R. pipiens* | YPM | 1143 |  | CT | New Haven | E,F,M |  |
| *R. pipiens* | YPM | 2906 |  | CT | New Haven | E,F,M |  |
| *R. pipiens* | YPM | 2907 |  | CT | New Haven | E,F,M |  |
| *R. pipiens* | YPM | 2908 |  | CT | New Haven | E,F,M |  |
| *R. pipiens* | YPM | 2909 |  | CT | New Haven | E,F,M |  |
| *R. pipiens* | YPM | 3068 |  | CT | New Haven | E,F,M |  |
| **Table S1.** | **Continued** |  |  |  |  |  |  |
| Taxon | Museum | Specimen No. | Notes | State/Province | County/Region | Analysis Type |  |
| *R. pipiens* | YPM | 3069 |  | CT | New Haven | E,F,M |  |
| *R. pipiens* | AMNH | 106549 |  | NY | Bronx | E |  |
| *R. pipiens* | AMNH | 106550 |  | NY | Bronx | E |  |
| *R. pipiens* | AMNH | 36651 | Long Island | NY | Queens | E |  |
| *R. pipiens* | YPM | 1045 |  | Quebec | Gaspe Peninsula | F,M |  |
| *R. pipiens* | YPM | 1048 |  | Quebec | Gaspe Peninsula | F,M |  |
| *R. pipiens* | YPM | 2965 |  | Quebec | Gaspe Peninsula | F,M |  |
| *R. pipiens* | YPM | 2966 |  | Quebec | Gaspe Peninsula | F,M |  |
| *R. pipiens* | YPM | 2967 |  | Quebec | Gaspe Peninsula | F,M |  |
| *R. pipiens* | YPM | 2968 |  | Quebec | Gaspe Peninsula | F,M |  |
| *R. pipiens* | YPM | 2969 |  | Quebec | Gaspe Peninsula | F,M |  |
| *R. pipiens* | YPM | 2970 |  | Quebec | Gaspe Peninsula | F,M |  |
| *R. pipiens* | YPM | 2971 |  | Quebec | Gaspe Peninsula | F,M |  |
| *R. pipiens* | YPM | 2972 |  | Quebec | Gaspe Peninsula | F,M |  |
| *R. pipiens* | YPM | 2973 |  | Quebec | Gaspe Peninsula | F,M |  |
| *R. pipiens* | YPM | 2976 |  | Quebec | Gaspe Peninsula | F,M |  |
| *R. pipiens* | YPM | 2977 |  | Quebec | Gaspe Peninsula | F,M |  |
| *R. pipiens* | YPM | 2978 |  | Quebec | Gaspe Peninsula | F,M |  |
| *R. pipiens* | YPM | 2979 |  | Quebec | Gaspe Peninsula | F,M |  |
| *R. pipiens* | YPM | 5808 |  | RI | Aquidneck Island | F,M |  |
| *R. pipiens* | YPM | 5809 |  | RI | Aquidneck Island | F,M |  |
| *R. pipiens* | YPM | 5810 |  | RI | Aquidneck Island | F,M |  |
| *R. sphenocephala* | YPM | 10485 |  | FL | Baker | M,R |  |
| *R. sphenocephala* | YPM | 1029 |  | FL | Miami-Dade | M |  |
| *R. sphenocephala* | YPM | 2930 |  | FL | Miami-Dade | R |  |
| *R. sphenocephala* | YPM | 4809 |  | FL | Miami-Dade | R |  |
| *R. sphenocephala* | YPM | 3082 |  | FL | Monroe | M,R |  |
| *R. sphenocephala* | YPM | 1023 |  | FL | Palm Beach | M,R |  |
| *R. sphenocephala* | YPM | 7320 |  | FL | Palm Beach | M,R |  |
| *R. sphenocephala* | YPM | 7321 |  | FL | Palm Beach | M,R |  |
| *R. sphenocephala* | YPM | 13805 |  | NC | Dare | C | † |
| *R. sphenocephala* | YPM | 13803 |  | NC | Dare | C,SN | † |
| *R. sphenocephala* | YPM | 13804 |  | NC | Dare | C,SN | † |
| **Table S1.** | **Continued** |  |  |  |  |  |  |
| Taxon | Museum | Specimen No. | Notes | State/Province | County/Region | Analysis Type |  |
| *R. sphenocephala* | ANSP | 3969 |  | NJ | Atlantic | M |  |
| *R. sphenocephala* | AMNH | 121144 |  | NJ | Burlington | F |  |
| *R. sphenocephala* | AMNH | 121145 |  | NJ | Burlington | F |  |
| *R. sphenocephala* | AMNH | 121148 |  | NJ | Burlington | F |  |
| *R. sphenocephala* | AMNH | 121149 |  | NJ | Burlington | F |  |
| *R. sphenocephala* | AMNH | 121151 |  | NJ | Burlington | F |  |
| *R. sphenocephala* | AMNH | 121153 |  | NJ | Burlington | F |  |
| *R. sphenocephala* | AMNH | 121154 |  | NJ | Burlington | F |  |
| *R. sphenocephala* | AMNH | 121155 |  | NJ | Burlington | F |  |
| *R. sphenocephala* | AMNH | 121156 |  | NJ | Burlington | F |  |
| *R. sphenocephala* | ANSP | 14939 |  | NJ | Burlington | M |  |
| *R. sphenocephala* | ANSP | 14947 |  | NJ | Burlington | M |  |
| *R. sphenocephala* | ANSP | 27110 |  | NJ | Burlington | M |  |
| *R. sphenocephala* | ANSP | 28819 |  | NJ | Burlington | M |  |
| *R. sphenocephala* | ANSP | 34478 |  | NJ | Burlington | M |  |
| *R. sphenocephala* | ANSP | 36804 |  | NJ | Burlington | M |  |
| *R. sphenocephala* | CM | 26238 |  | NJ | Burlington | M |  |
| *R. sphenocephala* | CM | 26242 |  | NJ | Burlington | M |  |
| *R. sphenocephala* | CM | 26243 |  | NJ | Burlington | M |  |
| *R. sphenocephala* | CM | 26244 |  | NJ | Burlington | M |  |
| *R. sphenocephala* | CM | 62005 |  | NJ | Burlington | M |  |
| *R. sphenocephala* | CM | 140094 |  | NJ | Burlington | M |  |
| *R. sphenocephala* | OMNH | 32414 |  | NJ | Burlington | M |  |
| *R. sphenocephala* | OMNH | 32415 |  | NJ | Burlington | M |  |
| *R. sphenocephala* | OMNH | 32416 |  | NJ | Burlington | M |  |
| *R. sphenocephala* | OMNH | 32417 |  | NJ | Burlington | M |  |
| *R. sphenocephala* | OMNH | 32418 |  | NJ | Burlington | M |  |
| *R. sphenocephala* | OMNH | 32419 |  | NJ | Burlington | M |  |
| *R. sphenocephala* | OMNH | 32420 |  | NJ | Burlington | M |  |
| *R. sphenocephala* | OMNH | 32421 |  | NJ | Burlington | M |  |
| *R. sphenocephala* | OMNH | 32422 |  | NJ | Burlington | M |  |
| *R. sphenocephala* | OMNH | 32423 |  | NJ | Burlington | M |  |
| *R. sphenocephala* | YPM | 13799 |  | NJ | Burlington | C | † |
| **Table S1.** | **Continued** |  |  |  |  |  |  |
| Taxon | Museum | Specimen No. | Notes | State/Province | County/Region | Analysis Type |  |
| *R. sphenocephala* | YPM | 13856 |  | NJ | Burlington | C | † |
| *R. sphenocephala* | YPM | 14061 |  | NJ | Burlington | C | † |
| *R. sphenocephala* | YPM | 14076 |  | NJ | Burlington | C | † |
| *R. sphenocephala* | YPM | 14080 |  | NJ | Burlington | C | † |
| *R. sphenocephala* | YPM | 14082 |  | NJ | Burlington | C | † |
| *R. sphenocephala* | YPM | 14083 |  | NJ | Burlington | C | † |
| *R. sphenocephala* | YPM | 14072 |  | NJ | Burlington | C,D,F,SH | † |
| *R. sphenocephala* | YPM | 14085 |  | NJ | Burlington | C,D,F,SH | † |
| *R. sphenocephala* | YPM | 14035 |  | NJ | Burlington | C,D,F,SH,SN |  |
| *R. sphenocephala* | YPM | 14039 |  | NJ | Burlington | C,D,F,SH,SN |  |
| *R. sphenocephala* | YPM | 14097 |  | NJ | Burlington | C,D,F,SH,SN | † |
| *R. sphenocephala* | YPM | 13771 |  | NJ | Burlington | C,D,SH,SN | † |
| *R. sphenocephala* | YPM | 13794 |  | NJ | Burlington | C,D,SH,SN | † |
| *R. sphenocephala* | YPM | 14058 |  | NJ | Burlington | C,D,SH,SN | † |
| *R. sphenocephala* | YPM | 14059 |  | NJ | Burlington | C,D,SH,SN | † |
| *R. sphenocephala* | YPM | 14064 |  | NJ | Burlington | C,D,SH,SN | † |
| *R. sphenocephala* | YPM | 14069 |  | NJ | Burlington | C,D,SH,SN | † |
| *R. sphenocephala* | YPM | 14070 |  | NJ | Burlington | C,D,SH,SN | † |
| *R. sphenocephala* | YPM | 14084 |  | NJ | Burlington | C,D,SH,SN | † |
| *R. sphenocephala* | YPM | 14089 |  | NJ | Burlington | C,D,SH,SN | † |
| *R. sphenocephala* | YPM | 14096 |  | NJ | Burlington | C,D,SH,SN | † |
| *R. sphenocephala* | YPM | 14102 |  | NJ | Burlington | C,D,SH,SN | † |
| *R. sphenocephala* | YPM | 14103 |  | NJ | Burlington | C,D,SH,SN | † |
| *R. sphenocephala* | YPM | 13769 |  | NJ | Burlington | C,F,SH,SN |  |
| *R. sphenocephala* | YPM | 13796 |  | NJ | Burlington | C,F,SH,SN | † |
| *R. sphenocephala* | YPM | 14034 |  | NJ | Burlington | C,F,SH,SN |  |
| *R. sphenocephala* | YPM | 14036 |  | NJ | Burlington | C,F,SH,SN |  |
| *R. sphenocephala* | YPM | 14037 |  | NJ | Burlington | C,F,SH,SN |  |
| *R. sphenocephala* | YPM | 14038 |  | NJ | Burlington | C,F,SH,SN |  |
| *R. sphenocephala* | YPM | 14040 |  | NJ | Burlington | C,F,SH,SN |  |
| *R. sphenocephala* | YPM | 14041 |  | NJ | Burlington | C,F,SH,SN |  |
| *R. sphenocephala* | YPM | 14042 |  | NJ | Burlington | C,F,SH,SN |  |
| *R. sphenocephala* | YPM | 14043 |  | NJ | Burlington | C,F,SH,SN |  |
| **Table S1.** | **Continued** |  |  |  |  |  |  |
| Taxon | Museum | Specimen No. | Notes | State/Province | County/Region | Analysis Type |  |
| *R. sphenocephala* | YPM | 14044 |  | NJ | Burlington | C,F,SH,SN |  |
| *R. sphenocephala* | YPM | 14045 |  | NJ | Burlington | C,F,SH,SN |  |
| *R. sphenocephala* | YPM | 14046 |  | NJ | Burlington | C,F,SH,SN |  |
| *R. sphenocephala* | YPM | 14047 |  | NJ | Burlington | C,F,SH,SN |  |
| *R. sphenocephala* | YPM | 14088 |  | NJ | Burlington | C,F,SH,SN | † |
| *R. sphenocephala* | YPM | 14105 |  | NJ | Burlington | C,F,SH,SN | † |
| *R. sphenocephala* | YPM | 14063 |  | NJ | Burlington | C,SH | † |
| *R. sphenocephala* | YPM | 14065 |  | NJ | Burlington | C,SH | † |
| *R. sphenocephala* | YPM | 14068 |  | NJ | Burlington | C,SH | † |
| *R. sphenocephala* | YPM | 14073 |  | NJ | Burlington | C,SH | † |
| *R. sphenocephala* | YPM | 14077 |  | NJ | Burlington | C,SH | † |
| *R. sphenocephala* | YPM | 14079 |  | NJ | Burlington | C,SH | † |
| *R. sphenocephala* | YPM | 14106 |  | NJ | Burlington | C,SH | † |
| *R. sphenocephala* | YPM | 13772 |  | NJ | Burlington | C,SH,SN | † |
| *R. sphenocephala* | YPM | 13795 |  | NJ | Burlington | C,SH,SN | † |
| *R. sphenocephala* | YPM | 13797 |  | NJ | Burlington | C,SH,SN | † |
| *R. sphenocephala* | YPM | 13800 |  | NJ | Burlington | C,SH,SN | † |
| *R. sphenocephala* | YPM | 13857 |  | NJ | Burlington | C,SH,SN | † |
| *R. sphenocephala* | YPM | 13859 |  | NJ | Burlington | C,SH,SN | † |
| *R. sphenocephala* | YPM | 13860 |  | NJ | Burlington | C,SH,SN | † |
| *R. sphenocephala* | YPM | 13861 |  | NJ | Burlington | C,SH,SN | † |
| *R. sphenocephala* | YPM | 14053 |  | NJ | Burlington | C,SH,SN | † |
| *R. sphenocephala* | YPM | 14054 |  | NJ | Burlington | C,SH,SN | † |
| *R. sphenocephala* | YPM | 14055 |  | NJ | Burlington | C,SH,SN | † |
| *R. sphenocephala* | YPM | 14056 |  | NJ | Burlington | C,SH,SN | † |
| *R. sphenocephala* | YPM | 14060 |  | NJ | Burlington | C,SH,SN | † |
| *R. sphenocephala* | YPM | 14066 |  | NJ | Burlington | C,SH,SN | † |
| *R. sphenocephala* | YPM | 14067 |  | NJ | Burlington | C,SH,SN | † |
| *R. sphenocephala* | YPM | 14071 |  | NJ | Burlington | C,SH,SN | † |
| *R. sphenocephala* | YPM | 14081 |  | NJ | Burlington | C,SH,SN | † |
| *R. sphenocephala* | YPM | 14090 |  | NJ | Burlington | C,SH,SN | † |
| *R. sphenocephala* | YPM | 14092 |  | NJ | Burlington | C,SH,SN | † |
| *R. sphenocephala* | YPM | 14093 |  | NJ | Burlington | C,SH,SN | † |
| **Table S1.** | **Continued** |  |  |  |  |  |  |
| Taxon | Museum | Specimen No. | Notes | State/Province | County/Region | Analysis Type |  |
| *R. sphenocephala* | YPM | 14100 |  | NJ | Burlington | C,SH,SN | † |
| *R. sphenocephala* | YPM | 13802 |  | NJ | Burlington | C,SN | † |
| *R. sphenocephala* | YPM | 13858 |  | NJ | Burlington | C,SN | † |
| *R. sphenocephala* | YPM | 14049 |  | NJ | Burlington | C,SN | † |
| *R. sphenocephala* | YPM | 14057 |  | NJ | Burlington | C,SN | † |
| *R. sphenocephala* | YPM | 14062 |  | NJ | Burlington | C,SN | † |
| *R. sphenocephala* | YPM | 14074 |  | NJ | Burlington | C,SN | † |
| *R. sphenocephala* | YPM | 14075 |  | NJ | Burlington | C,SN | † |
| *R. sphenocephala* | YPM | 14078 |  | NJ | Burlington | C,SN | † |
| *R. sphenocephala* | YPM | 14087 |  | NJ | Burlington | C,SN | † |
| *R. sphenocephala* | YPM | 14091 |  | NJ | Burlington | C,SN | † |
| *R. sphenocephala* | YPM | 14094 |  | NJ | Burlington | C,SN | † |
| *R. sphenocephala* | YPM | 14095 |  | NJ | Burlington | C,SN | † |
| *R. sphenocephala* | YPM | 14098 |  | NJ | Burlington | C,SN | † |
| *R. sphenocephala* | YPM | 14099 |  | NJ | Burlington | C,SN | † |
| *R. sphenocephala* | YPM | 14101 |  | NJ | Burlington | C,SN | † |
| *R. sphenocephala* | YPM | 14104 |  | NJ | Burlington | C,SN | † |
| *R. sphenocephala* | YPM | 13770 |  | NJ | Burlington | SH,SN | †† |
| *R. sphenocephala* | ANSP | 19286 |  | NJ | Camden | M |  |
| *R. sphenocephala* | ANSP | 15852 |  | NJ | Cape May | M |  |
| *R. sphenocephala* | ANSP | 17606 |  | NJ | Cape May | M |  |
| *R. sphenocephala* | ANSP | 17607 |  | NJ | Cape May | M |  |
| *R. sphenocephala* | ANSP | 17608 |  | NJ | Cape May | M |  |
| *R. sphenocephala* | ANSP | 17609 |  | NJ | Cape May | M |  |
| *R. sphenocephala* | ANSP | 17610 |  | NJ | Cape May | M |  |
| *R. sphenocephala* | AMNH | 116842 |  | NJ | Middlesex | F |  |
| *R. sphenocephala* | YPM | 14030 |  | NJ | Middlesex | C,F,SH,SN | † |
| *R. sphenocephala* | YPM | 14031 |  | NJ | Middlesex | C,SN | † |
| *R. sphenocephala* | ANSP | 16394 |  | NJ | Ocean | M |  |
| *R. sphenocephala* | ANSP | 16395 |  | NJ | Ocean | M |  |
| *R. sphenocephala* | CM | 11593 |  | NJ | Ocean | M |  |
| *R. sphenocephala* | CM | 11594 |  | NJ | Ocean | M |  |
| *R. sphenocephala* | CM | 11595 |  | NJ | Ocean | M |  |
| **Table S1.** | **Continued** |  |  |  |  |  |  |
| Taxon | Museum | Specimen No. | Notes | State/Province | County/Region | Analysis Type |  |
| *R. sphenocephala* | CM | 11596 |  | NJ | Ocean | M |  |
| *R. sphenocephala* | CM | 28578 |  | NJ | Ocean | M |  |
| *R. sphenocephala* | CM | 28579 |  | NJ | Ocean | M |  |
| *R. sphenocephala* | CM | 39717 |  | NJ | Ocean | M |  |
| *R. sphenocephala* | OMNH | 30537 |  | NJ | Ocean | M |  |
| *R. sphenocephala* | YPM | 13801 |  | NJ | Ocean | C,D,F,SH,SN | † |
| *R. sphenocephala* | YPM | 14051 |  | NJ | Ocean | C,F | † |
| *R. sphenocephala* | YPM | 13798 |  | NJ | Ocean | C,F,SH,SN | † |
| *R. sphenocephala* | YPM | 14050 |  | NJ | Ocean | C,SH,SN | † |
| *R. sphenocephala* | YPM | 14052 |  | NJ | Ocean | C,SH,SN | † |
| *R. sphenocephala* (tentative) | AMNH | 125956 | Long Island; further examination needed | NY | Suffolk | E |  |
| *R. sphenocephala* (tentative) | AMNH | 176153 | Long Island; further examination needed | NY | Suffolk | E |  |
