# Supplementary material for: Cryptic Diversity in Metropolis: Confirmation of a New Leopard Frog Species (Anura: Ranidae) from New York City and Surrounding Atlantic Coast Regions
Source: PLoS One. 2014 Oct 29;9(10):e108213. doi: 10.1371/journal.pone.0108213 (PMC4212910; doi:10.1371/journal.pone.0108213)
Supplement: Table S2 — List of Rana primary (advertisement) calls measured for bioacoustic data. (DOC) [file pone.0108213.s006.doc]

| **Table S2.** List of *Rana* primary (advertisement) calls measured for bioacoustic data. All recordings are deposited at Yale Peabody Museum (YPM). Localities are listed by state, county, and city (or region, when more specific). Additional locality information is available from authors. Frog number 4 was omitted from analysis. Track time is provided for recordings with multiple analyzed frogs to differentiate individuals using the start time of their respective call series. * = Recorded with Olympus LS-1X series linear PCM recorder (at sample rate of 99.6 kHz and sample size of 24-bits). Each frog contributed call-attributes based on average values derived from four successive calls unless otherwise noted († = three successive calls, § = two successive calls). Frogs 15-18 recorded by E. Kiviat, frogs 23-26 by BRC, and frog 27 by S. Amburgey. All other frogs recorded by JAF. | | | | | | | | | |
| --- | --- | --- | --- | --- | --- | --- | --- | --- | --- |
| Taxon | Frog No. | YPM Catalog No. | State | County | City or Region | Population | Water Temp. (°C) | Track Time (s) |  |
| *R. kauffeldi* | 1 | 14137, 14172 | NY | Richmond | Bloomfield | 1 | 10.0 |  |  |
| *R. kauffeldi* | 2 | 14138 | NY | Richmond | Bloomfield | 1 | 10.0 |  |  |
| *R. kauffeldi* | 3 | 14139 | NY | Richmond | Bloomfield | 1 | 10.0 |  |  |
| *R. kauffeldi* | 5 | 14140 | NY | Richmond | Bloomfield | 1 | 10.0 | 7.35 |  |
| *R. kauffeldi* | 6 | 14140 | NY | Richmond | Bloomfield | 1 | 10.0 | 7.73 |  |
| *R. kauffeldi* | 7 | 14141 | NY | Richmond | Bloomfield | 1 | 11.9 | 7.14 |  |
| *R. kauffeldi* | 8 | 14141 | NY | Richmond | Bloomfield | 1 | 11.9 | 16.31 |  |
| *R. kauffeldi* | 9 | 14142 | NY | Richmond | Bloomfield | 1 | 11.9 |  |  |
| *R. kauffeldi* | 10 | 14143 | NJ | Bergen | Moonachie | 2 | 17.2 | 7.84 |  |
| *R. kauffeldi* | 11 | 14143 | NJ | Bergen | Moonachie | 2 | 17.2 | 23.36 |  |
| *R. kauffeldi* | 12 | 14144 | NJ | Bergen | Moonachie | 2 | 17.2 |  |  |
| *R. kauffeldi* | 13 | 14145 | NY | Richmond | Bloomfield | 3 | 13.0 | 0.31 |  |
| *R. kauffeldi* | 14 | 14145 | NY | Richmond | Bloomfield | 3 | 13.0 | 10.30 |  |
| *R. pipiens* | 15 | 14163 | NY | Columbia | Hudson | 1 | 18.0 | 8.37 | * |
| *R. pipiens* | 16 | 14163 | NY | Columbia | Hudson | 1 | 18.0 | 6.20 | *† |
| *R. pipiens* | 17 | 14163 | NY | Columbia | Hudson | 1 | 18.0 | 16.06 | * |
| *R. pipiens* | 18 | 14163 | NY | Columbia | Hudson | 1 | 18.0 | 53.44 | *† |
| *R. sphenocephala* | 19 | 14146 | NJ | Middlesex | South Brunswick | 1 | 11.0 | 9.31 |  |
| *R. sphenocephala* | 20 | 14147 | NJ | Middlesex | South Brunswick | 1 | 11.0 |  |  |
| *R. sphenocephala* | 21 | 14146 | NJ | Middlesex | South Brunswick | 1 | 11.0 | 4.03 |  |
| *R. sphenocephala* | 22 | 14148 | NJ | Middlesex | South Brunswick | 1 | 11.0 |  |  |
| *R. sphenocephala* | 23 | 14149 | NJ | Burlington | New Gretna | 2 | 25.6 | 32.79 | * |
| *R. sphenocephala* | 24 | 14150 | NJ | Burlington | New Gretna | 2 | 25.6 | 5.08 | * |
| *R. sphenocephala* | 25 | 14149 | NJ | Burlington | New Gretna | 2 | 25.6 | 2.27 | * |
| *R. sphenocephala* | 26 | 14150 | NJ | Burlington | New Gretna | 2 | 25.6 | 13.26 | *† |
| *R. sylvatica* | 27 | 14151 | CO | Larimer | Cameron Pass | 1 | 11.0 |  |  |
| *R. sylvatica* | 28 | 14152 | NY | Suffolk | Upton | 2 | 10.1 | 13.90 |  |

| **Table S2. Continued** | | | | | | | | |  | |
| --- | --- | --- | --- | --- | --- | --- | --- | --- | --- | --- |
| Taxon | Frog No. | YPM Catalog No. | State | County | City or Region | Population | Water Temp. (°C) | Track Time (s) | |  |
| *R. sylvatica* | 29 | 14152 | NY | Suffolk | Upton | 2 | 10.1 | 7.27 | |  |
| *R. sylvatica* | 30 | 14152 | NY | Suffolk | Upton | 2 | 10.1 | 4.53 | | † |
| *R. sylvatica* | 31 | 14152 | NY | Suffolk | Upton | 2 | 10.1 | 18.35 | |  |
| *R. sylvatica* | 32 | 14152 | NY | Suffolk | Upton | 2 | 10.1 | 35.08 | |  |
| *R. sylvatica* | 33 | 14153 | NY | Queens | Alley Park | 3 | 8.0 |  | |  |
| *R. sylvatica* | 34 | 14154 | NY | Queens | Alley Park | 3 | 8.8 | 26.15 | |  |
| *R. sylvatica* | 35 | 14154 | NY | Queens | Alley Park | 3 | 8.8 | 2.83 | |  |
| *R. palustris* | 36 | 14155 | NY | Suffolk | Calverton | 1 | 15.0 |  | | § |
| *R. palustris* | 37 | 14156 | NY | Suffolk | Calverton | 1 | 15.0 |  | | § |
| *R. palustris* | 38 | 14157 | NY | Suffolk | Calverton | 1 | 15.0 |  | |  |
| *R. palustris* | 39 | 14158 | NY | Suffolk | Calverton | 1 | 15.0 |  | |  |
| *R. palustris* | 40 | 14159 | NY | Suffolk | Calverton | 1 | 15.0 | 2.42 | | † |
| *R. palustris* | 41 | 14159 | NY | Suffolk | Calverton | 1 | 15.0 | 5.22 | |  |
| *R. palustris* | 42 | 14160 | NY | Suffolk | Calverton | 1 | 15.0 |  | | † |
| *R. palustris* | 43 | 14161 | NY | Suffolk | Calverton | 1 | 15.0 | 1.13 | | † |
| *R. palustris* | 44 | 14161 | NY | Suffolk | Calverton | 1 | 15.0 | 2.24 | | † |
| *R. palustris* | 45 | 14162 | NY | Suffolk | Calverton | 1 | 15.0 | 11.86 | | † |
| *R. palustris* | 46 | 14162 | NY | Suffolk | Calverton | 1 | 15.0 | 4.23 | | † |
