# Supplementary material for: Cryptic Diversity in Metropolis: Confirmation of a New Leopard Frog Species (Anura: Ranidae) from New York City and Surrounding Atlantic Coast Regions
Source: PLoS One. 2014 Oct 29;9(10):e108213. doi: 10.1371/journal.pone.0108213 (PMC4212910; doi:10.1371/journal.pone.0108213)
Supplement: Table S3 — Classification matrix for four Rana species using discriminant function analysis on morphometric variables. (DOC) [file pone.0108213.s007.doc]

| **Table S3.** Classification matrix for four *Rana* species using discriminant function analysis on morphometric variables. | | | | |
| --- | --- | --- | --- | --- |
|  | Pairwise | | | |
| Original | *kauffeldi* | *sphenocephala* | *pipiens* | *palustris* |
| *kauffeldi* | 143 | 6 | 1 | 7 |
| *sphenocephala* | 15 | 31 | 0 | 0 |
| *pipiens* | 3 | 1 | 24 | 3 |
| *palustris* | 16 | 0 | 6 | 8 |
