# Supplementary material for: Cryptic Diversity in Metropolis: Confirmation of a New Leopard Frog Species (Anura: Ranidae) from New York City and Surrounding Atlantic Coast Regions
Source: PLoS One. 2014 Oct 29;9(10):e108213. doi: 10.1371/journal.pone.0108213 (PMC4212910; doi:10.1371/journal.pone.0108213)
Supplement: Table S4 — Coefficients for three discriminant functions (from four species of Rana ) for each of 12 morphological characters: head length (HL), head width (HW), eye diameter (ED), tympanum diameter (TD), foot length (FOL), eye-to-naris distance (END), naris-to-snout distance (NSD), thigh length (THL), internarial distance (IND), interorbital distance (IOD), shank length (SL), and dorsal snout angle (DSA). (DOC) [file pone.0108213.s008.doc]

| **Table S4.** Coefficients for three discriminant functions (from four species of Rana) for each of 12 morphological characters: head length (HL), head width (HW), eye diameter (ED), tympanum diameter (TD), foot length (FOL), eye-to-naris distance (END), naris-to-snout distance (NSD), thigh length (THL), internarial distance (IND), interorbital distance (IOD), shank length (SL), and dorsal snout angle (DSA). | | | |
| --- | --- | --- | --- |
|  | LD1 | LD2 | LD3 |
| HL | -0.464 | -1.098 | -1.293 |
| HW | -0.007 | 1.018 | 0.859 |
| ED | 0.512 | -0.119 | 0.739 |
| TD | -1.001 | -0.315 | 0.65 |
| FOL | -0.038 | -0.051 | 0.103 |
| END | 0.035 | -1.139 | -0.123 |
| NSD | 0.528 | 0.191 | 0.597 |
| THL | 0.034 | -0.028 | -0.133 |
| IND | -0.052 | 0.682 | -0.441 |
| IOD | -0.546 | 0.017 | 0.215 |
| SL | 0.375 | 0.238 | -0.212 |
| DSA | -2.02 | -8.427 | -17.234 |
