# Supplementary material for: Cryptic Diversity in Metropolis: Confirmation of a New Leopard Frog Species (Anura: Ranidae) from New York City and Surrounding Atlantic Coast Regions
Source: PLoS One. 2014 Oct 29;9(10):e108213. doi: 10.1371/journal.pone.0108213 (PMC4212910; doi:10.1371/journal.pone.0108213)
Supplement: Table S5 — Classification matrix for five Rana species using discriminant function analysis on bioacoustic variables. (DOC) [file pone.0108213.s009.doc]

| **Table S5.** Classification matrix for five *Rana* species using discriminant function analysis on bioacoustic | | | | | |
| --- | --- | --- | --- | --- | --- |
| variables. | | | | | |
|  | Pairwise | | | | |
| Original | *kauffeldi* | *sphenocephala* | *pipiens* | *palustris* | *sylvatica* |
| *kauffeldi* | 13 | 0 | 0 | 0 | 0 |
| *sphenocephala* | 0 | 8 | 0 | 0 | 0 |
| *pipiens* | 0 | 0 | 4 | 0 | 0 |
| *palustris* | 0 | 0 | 0 | 11 | 0 |
| *sylvatica* | 2 | 0 | 0 | 0 | 7 |
