# Supplementary material for: Cryptic Diversity in Metropolis: Confirmation of a New Leopard Frog Species (Anura: Ranidae) from New York City and Surrounding Atlantic Coast Regions
Source: PLoS One. 2014 Oct 29;9(10):e108213. doi: 10.1371/journal.pone.0108213 (PMC4212910; doi:10.1371/journal.pone.0108213)
Supplement: Table S6 — Coefficients for four discriminant functions (from five species of Rana ) for each of six bioacoustic characters: call length (CL), call rate (CR), call rise time (CRT), call duty cycle (CDC), pulse number (PN), and dominant frequency (DF). (DOC) [file pone.0108213.s010.doc]

| **Table S6.** Coefficients for four discriminant functions (from five species of *Rana*) for each of six bioacoustic characters: call length (CL), call rate (CR), call rise time (CRT), call duty cycle (CDC), pulse number (PN), and dominant frequency (DF). | | | | |
| --- | --- | --- | --- | --- |
|  | LD1 | LD2 | LD3 | LD4 |
| CL | 1.808 | 7.698 | 2.16 | -1.97 |
| CR | -0.605 | -0.779 | 1.187 | -1.039 |
| CRT | -2.806 | -1.751 | 0.964 | 4.409 |
| CDC | 1.263 | 4.855 | -8.933 | -3.346 |
| PN | 0.185 | -0.199 | -0.023 | -0.057 |
| DF | 0 | -0.003 | 0.001 | -0.001 |
